# A Functional Screening Identifies a New Organic Selenium Compound Targeting Cancer Stem Cells: Role of c‐Myc Transcription Activity Inhibition in Liver Cancer

**DOI:** 10.1002/advs.202201166

**Published:** 2022-06-02

**Authors:** Jun‐Nian Zhou, Biao Zhang, Hai‐Yang Wang, Dong‐Xing Wang, Ming‐Ming Zhang, Min Zhang, Xiao‐Kui Wang, Shi‐Yong Fan, Ying‐Chen Xu, Quan Zeng, Ya‐Li Jia, Jia‐Fei Xi, Xue Nan, Li‐Juan He, Xin‐Bo Zhou, Song Li, Wu Zhong, Wen Yue, Xue‐Tao Pei

**Affiliations:** ^1^ Stem Cell and Regenerative Medicine Lab Beijing Institute of Radiation Medicine Beijing 100850 P. R. China; ^2^ South China Research Center for Stem Cell and Regenerative Medicine SCIB Guangzhou 510005 P. R. China; ^3^ National Engineering Research Center for the Emergency Drug Beijing Institute of Pharmacology and Toxicology Beijing 100850 P. R. China; ^4^ Department of Hepatobiliary Surgery Beijing Tongren Hospital Beijing 100730 P. R. China

**Keywords:** chemoresistance, c‐Myc, hepatocellular carcinoma, liver cancer stem cells, metastasis, selenium

## Abstract

Cancer stem cells (CSCs) are reported to play essential roles in chemoresistance and metastasis. Pathways regulating CSC self‐renewal and proliferation, such as Hedgehog, Notch, Wnt/*β*‐catenin, TGF‐*β*, and Myc, may be potential therapeutic targets. Here, a functional screening from the focused library with 365 compounds is performed by a step‐by‐step strategy. Among these candidate molecules, phenyl‐2‐pyrimidinyl ketone 4‐allyl‐3‐amino selenourea (CU27) is chosen for further identification because it proves to be the most effective compound over others on CSC inhibition. Through ingenuity pathway analysis, it is shown CU27 may inhibit CSC through a well‐known stemness‐related transcription factor c‐Myc. Gene set enrichment analysis, dual‐luciferase reporter assays, expression levels of typical c‐Myc targets, molecular docking, surface plasmon resonance, immunoprecipitation, and chromatin immunoprecipitation are conducted. These results together suggest CU27 binds c‐Myc bHLH/LZ domains, inhibits c‐Myc‐Max complex formation, and prevents its occupancy on target gene promoters. In mouse models, CU27 significantly sensitizes sorafenib‐resistant tumor to sorafenib, reduces the primary tumor size, and inhibits CSC generation, showing a dramatic anti‐metastasis potential. Taken together, CU27 exerts inhibitory effects on CSC and CSC‐associated traits in hepatocellular carcinoma (HCC) via c‐Myc transcription activity inhibition. CU27 may be a promising therapeutic to treat sorafenib‐resistant HCC.

## Graphical abstract

A novel organic selenium compound, phenyl‐2‐pyrimidinyl ketone 4‐allyl‐3‐amino selenourea (CU27), is developed, which can bind c‐Myc bHLH/LZ domains, block c‐Myc‐Max complex formation, and prevent its occupancy on target gene promoters in hepatocellular carcinoma (HCC) cells. These effects lead to the suppression of cancer stem cell self‐renewal, resulting in inhibition of metastasis and sorafenib resistance in HCC. CU27 may be a promising therapeutic to treat sorafenib‐resistant HCC.

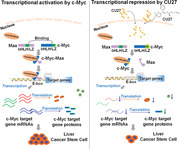

## 1 Introduction

Hepatocellular carcinoma (HCC) is the most common cancer type of liver cancer which causes nearly 600 000 deaths annually.^[^ ^1^ ^]^ Like in most advanced cancers, high rate of metastasis, recurrence, and limited therapeutic options are considered to be responsible for the poor prognosis of HCC.^[^ ^2^ ^]^ A growing body of evidence suggests a subpopulation of cancer cells with self‐renewing and tumor‐initiating capacity, known as CSCs,^[^ ^3^ ^]^ have pivotal roles in tumor relapse and distant organ metastasis.

The CSC concept has been adopted in preclinical drug discovery programs; However, challenges of CSC‐targeted drug discovery including CSC heterogeneity, at both inter‐ and intratumor levels, and selectivity over endogenous stem cells complicate the drug discovery efforts.^[^ ^4^ ^]^ So far, approaches based on targeting the essential pathways or molecules that are reported to regulate stemness, to prevent and/or treat metastasis, are thus emerging as a promising therapeutic strategy. Some stem‐cell regulators, such as Hedgehog, Notch, Wnt/*β*‐catenin pathway,^[^ ^5^, ^6^ ^]^ TGF‐*β* pathway^[^ ^7^ ^]^ and Myc,^[^ ^8^, ^9^ ^]^ which are essential for self‐renewal and proliferation of stem cell, are thus ideal potential therapeutic targets to prevent and treat cancer metastasis.

Among these potential therapeutic targets, c‐Myc is one of the “most wanted” targets for cancer therapy. c‐Myc is a highly amplified oncogene, being over‐expressed in up to 75% of cancers, has been found to play important roles in tumor progression and maintenance.^[^ ^10^ ^]^ Myc amplification is considered a driver mutation in many cancer types.^[^ ^11^ ^]^ Heterodimers formed by c‐Myc and MAX regulate downstream targets involved in proliferation, apoptosis, differentiation, and many other key biological processes in cancer cells.^[^ ^12^ ^]^ In liver cancer, an activated Myc transcription signature has been identified to be associated with the malignant conversion of pre‐neoplastic liver lesions.^[^ ^13^ ^]^ While inactivation of Myc in liver cancer cells can initiate a differentiating process of tumor cells into hepatocytes and biliary cells.^[^ ^14^ ^]^ It is reported c‐Myc is a key activator in reprogramming adult hepatocytes into CSCs,^[^ ^15^ ^]^ suggesting it may be a master regulator and an ideal potential target of liver CSCs.

However, c‐Myc is currently considered as an ‘undruggable’ or a ‘difficult to drug’ protein owing to its large protein–protein interaction network and lack of protein binding pockets for drugs.^[^ ^16^ ^]^ Despite efforts to target c‐Myc by different strategies, such as directly inhibiting c‐Myc expression by antisense^[^ ^17^ ^]^ or small molecules,^[^ ^18^ ^]^ interrupting the interactions among Myc‐Max‐DNA,^[^ ^19^ ^]^ and indirectly interfering with c‐Myc‐associated regulators,^[^ ^20^, ^21^ ^]^ up to now, identifying an approach to pharmacologically target c‐Myc is still one of the key challenges in cancer research. Thus, novel CSC inhibitors with c‐Myc‐suppressed potentials would be of great clinical significance for HCC patients.

In this project, we first made efforts in developing compounds with anti‐liver CSC potential from our designed pool including small molecules targeting mammalian target of rapamycin (mTOR) and protein kinase B (Akt), which is recently reported to be crucial for c‐Myc to promote hepato‐carcinogenesis,^[^ ^22^ ^]^ and organic selenium compounds and their sulfur‐containing analogues (referred to as CU), both of which have been reported to have antitumor potential.^[^ ^23^ ^]^ By means of a step‐by‐step functional screening strategy, we identified phenyl‐2‐pyrimidinyl ketone 4‐allyl‐3‐amino selenourea (referred to as CU27), with a most significant CSC‐inhibited potential. Next, we validated CU27 diminished liver CSCs‐associated phenotypes and functions in vitro and in vivo. Furthermore, we demonstrated that CU27 interrupts Myc/MAX complex formation and inhibits c‐Myc target genes transcription, suggesting its regulatory roles were mediated by c‐Myc transcription inhibition. Most of all, we found the sorafenib‐sensitization and anti‐metastasis role of CU27 in mouse model, showing a promising therapeutic potential of CU27 for HCC patients.

## 2 Results

### 2.1 Functional Screening Identified CU27, a Novel Organic‐Selenium Compound, Has CSC‐Inhibiting Potential in HCC Cells

**Figure 1:**
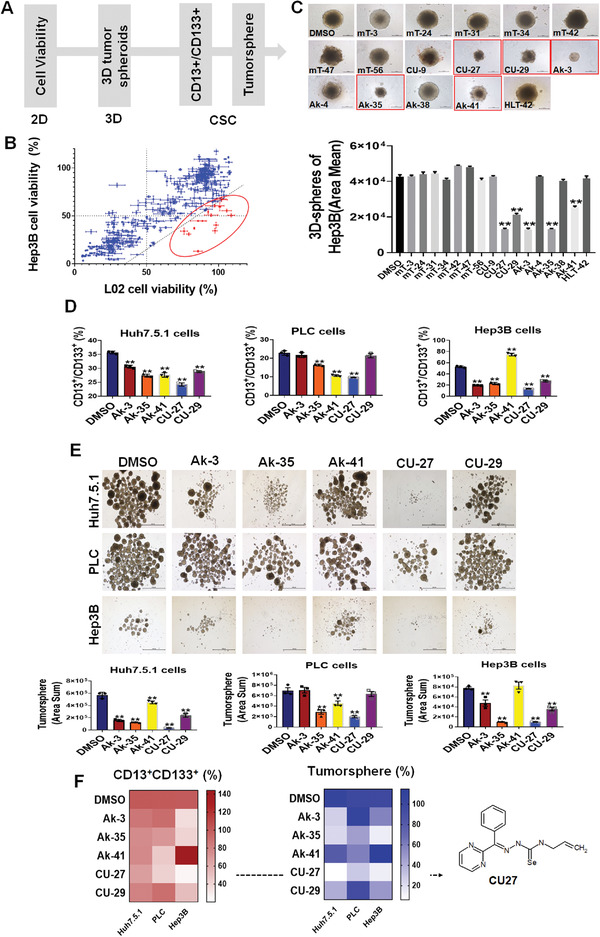
Functional screening of small molecules with a step‐by‐step strategy. A) Flowchart of functional screening. B) Scattered plot showing the cell viability of each small molecule in human normal fetal liver cell L02 and liver cancer Hep3B cells, 48 h after treatment with each compound (10 µm). Relative cell viability (L02 minus Hep3B) ≥35% as the screening criteria. *n* = 3, Means ± SD. C) Images and statistical histogram of 3D tumor spheroids from Hep3B (cultured for 5 days, followed by treated with 16 candidate compounds (10 µm) for another 3 days). Tumor spheroid was quantified as the total cross‐sectional area of spheroid in each group (area mean), *n* = 3, Means ± SD, ^*^ *p* < 0.05, ^**^ *p* < 0.01, Student's *t*‐test. D) Statistical histogram of the CD13^+^CD133^+^ population, 48 h after treatment with each candidate compound (10 µm), analyzed by FCM, *n* = 3, Means ± SD; ^*^ *p* < 0.05, ^**^ *p* < 0.01; Student's *t*‐test. E) Representative images of tumorspheres from HCC cells that received a 48‐h pretreatment with each candidate compound (10 µm) before tumorsphere culture. Bars, 1000 µm. Tumorspheres were quantified as the total cross‐sectional area of tumorspheres in each group (area sum), and similarly hereafter unless indicated. Statistical histogram of the tumorsphere size. *n* = 3, means ± SD, each compound compared with DMSO, ^*^ *p* < 0.05, ^**^ *p* < 0.01, Student's *t*‐test. F) Heat maps of functional screening results, shown by the relative value of CD13^+^CD133^+^ population (D) and tumorsphere size (E), with normalizing the vehicle group as 100%. CU27 chemical structure is shown in the right.

In order to search for novel CSC‐inhibiting small molecules, we have built a focused library with 365 compounds including targeting mTOR and Akt; thiosemicarbazones and selenosemicarbazones which are synthesized by ourselves (Figure S1, Supporting Information). Given that the common concentration used in in vitro drug screening is 10 µm,^[^ ^24^ ^]^ therefore, in the three rounds of drug screening, all small molecules are 10 µm. As shown in **Figure** **1**A, in the first step, we performed a relative cell viability‐based screening in the 2D‐cell‐culture model to remove candidate compounds that are more toxic to normal cells (Table S1, Supporting Information), and 15 candidate compounds were obtained (Figure 1B). Then we continued screening in the 3D‐cell‐culture model and obtained five compounds that significantly inhibit tumor spheroid size (Figure 1C). We further performed anti‐CSC screening on these five compounds through liver CSC population analysis (Figure 1D; Figure S2, Supporting Information) and tumorsphere formation (Figure 1E), a system that enriches CSC,^[^ ^25^ ^]^ as we previously reported,^[^ ^26^ ^]^ among which CU27 is most effective in three tested cell lines (Figure 1F).

Together, in our functional screening, CU27 proved to be the most effective compound over other compounds on CSC inhibition, thus we selected CU27 for further investigation.

### 2.2 CU27 Diminishes Liver CSC‐Associated Phenotypes and Functions Both In Vitro and In Vivo

**Figure 2:**
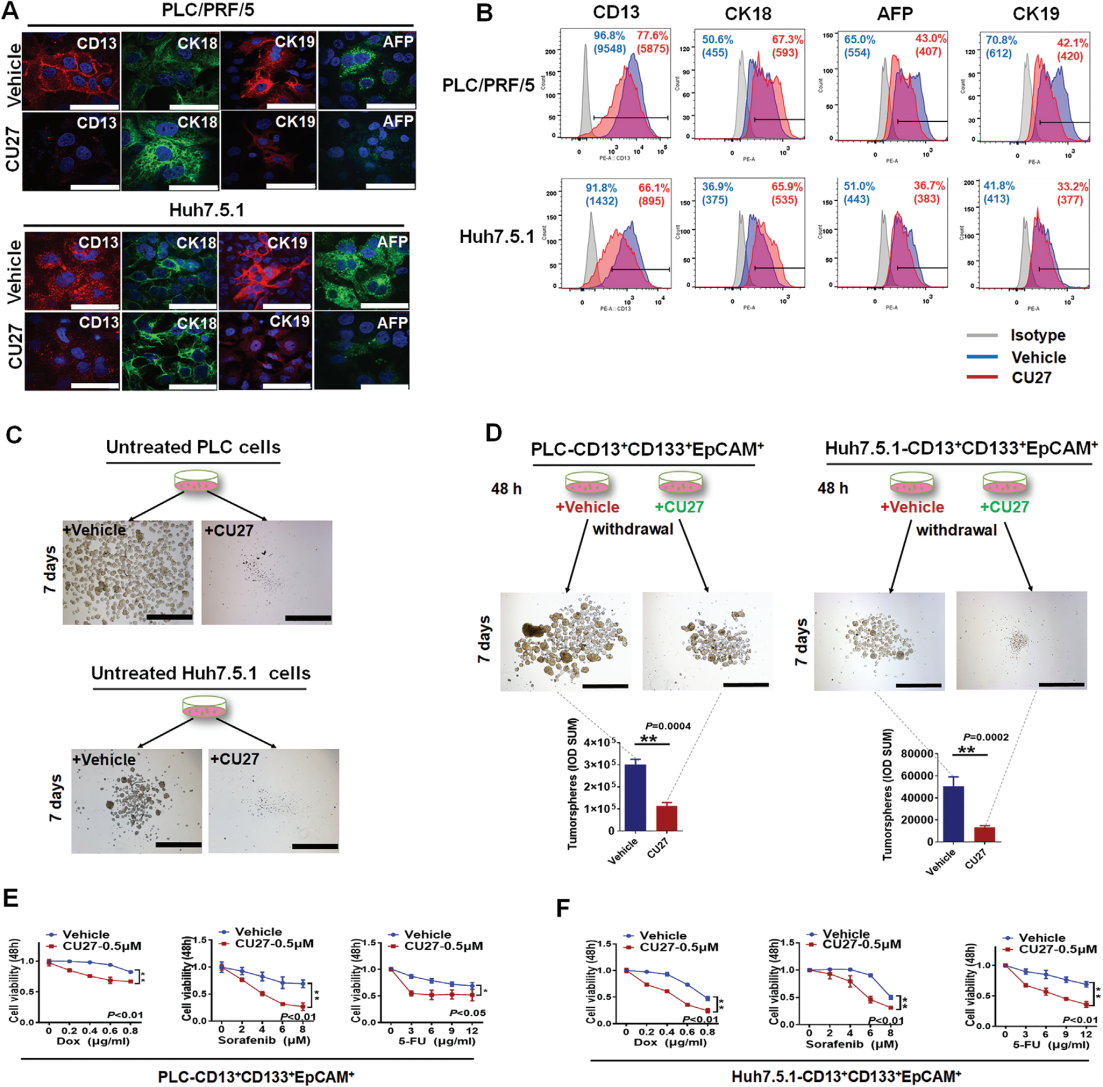
CU27 diminishes liver CSC‐associated phenotypes and functions in vitro. A,B) Representative immunofluorescence images and flow cytometry analysis of expression levels of CK18, CK19, and AFP besides CD13 in CU27‐treated HCC cells (10 µm, 48 h, *n* = 3). Bars, 100 µm. C) Representative images of untreated HCC cells‐derived tumorspheres simultaneously treated with CU27 (7 days). Bars, 1000 µm. D) Representative images and quantitative analysis of tumorspheres from sorted CD13^+^/CD133^+^/EpCAM^+^ HCC cells after a pre‐treatment with CU27 (48 h) in CDM culture before proceeding to tumorsphere formation. Bars, 1000 µm. Mean ± SD, ^**^ *p* < 0.01, data analyzed by Student's *t*‐test. E,F) Chemoresistance assay of doxorubicin (Dox), sorafenib, and 5‐Fluorouracil (5‐FU) in sorted CD13^+^/CD133^+^/EpCAM^+^ HCC cells treated with CU27 (0.5 µm) in CDM culture (IC_20_ of CU27, 48 h, *n* = 3). IC_20_, 20% growth inhibition concentration of CU27; Means ± SD; ^*^ *p* < 0.05, ^**^ *p* < 0.01; two‐way ANOVA.

We first made the proliferation dose‐response curves of CU27. The half maximal inhibitory concentration (IC_50_) value of CU27 in HCC cell line Hep3B was 5.24 ± 1.12 µm, while the IC_50_ value was 23.98 ± 1.50 µm in L02 (Figure S3, Supporting Information). Since CU27 does not significantly induce HCC cell apoptosis (Figure S4, Supporting Information), we performed an immunofluorescence staining and flow cytometry (FCM) to analyze the cell differentiation status after CU27‐treatment. We found the decreased expression levels of Alpha Fetoprotein (AFP), Cytokeratin 19 (CK19), and Aminopeptidase N (CD13) and an increased Cytokeratin 18 (CK18) expression in CU27‐treated cells compared with controls (**Figure** **2**A,B; Figure S5A,B, Supporting Information), indicating a well differentiation status.^[^ ^27^ ^]^ We have added CU27 and HCC cells together into the 3D tumorsphere system, and found that no tumorspheres were formed after 7‐days‐treatment of CU27 (Figure 2C; Figure S5C, Supporting Information). To address whether CU27 specifically targets CSCs, we isolated CD13^+^/Prominin‐1(CD133)^+^/Epithelial cell adhesion molecule(EpCAM)^+^ cells as the enriched liver CSCs.^[^ ^28^ ^]^ After a pre‐treatment with CU27 (48 h) in chemically defined medium (CDM) culture, the CSCs were then proceeded to tumorsphere formation. We demonstrated CU27 suppressed 1st tumorsphere formation (Figure 2D; Figure S5D, Supporting Information) and serial tumorsphere formation (Figure S6, Supporting Information). These results suggest CU27 specifically targets liver CSCs, possibly by promoting CSC differentiation in vitro.

As chemoresistance is also a hallmark of CSC, we investigated whether CU27 could sensitize CSCs to chemotherapeutics. Cell Counting Kit‐8 (CCK‐8) analysis showed a significant reduction in viability of CSC cells exposed to combination‐treatments with CU27, compared with single‐drug‐treatments (Figure 2E,F; Figure S5E, Supporting Information), suggesting synergy effects between CU27 and these chemotherapies on CSCs.

**Figure 3:**
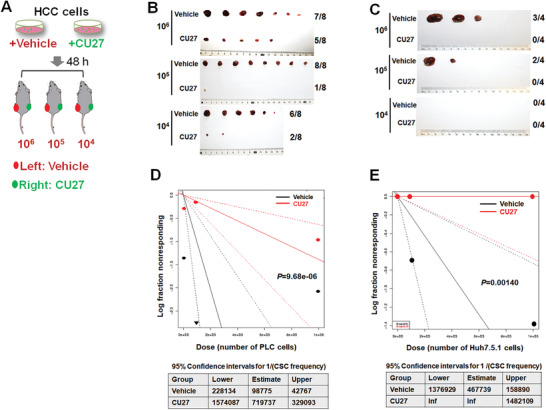
In vivo limiting dilution assay of CU27‐treated HCC cells. A) Schematic diagram of in vivo limiting dilution assay. B,C) PLC cells‐derived (B) and Huh7.5.1 cells‐derived (C) tumors dissected from two flanks of xenografted mice. D.E) Liver CSC frequency analysis by extreme limiting dilution analysis (ELDA) software.^[^ ^29^ ^]^ A plot of the log proportion of nonresponding versus the number of cells, the slope of the line representing the estimated log‐liver CSC fraction and the dotted lines give the 95% confidence interval. The value with zero negative response at dose 100 000 in vehicle is represented by a down‐pointing triangle (top). The estimated frequency and its 95% confidence intervals displayed in table (bottom).

We then explored whether CU27 can alter the in vivo tumor‐initiating capacity of CSCs by limiting dilution assay (**Figure** **3**A), which is the current gold‐standard for defining self‐renewal of CSCs in vivo.^[^ ^26^ ^]^ The tumor incidence at all three dilutions tested (Figure 3B,C) and the CSC frequencies of CU27‐pretreated cells (Figure 3D,E) was significantly lower than vehicle‐treated cells, suggesting CU27 inhibits the in vivo tumor‐initiating potential of HCC cells.

These results together demonstrate CU27 diminishes liver CSC‐associated phenotypes and functions both in vitro and in vivo.

### 2.3 CU27 Inhibits the Transcriptional Activity of c‐Myc in HCC Cells

**Figure 4:**
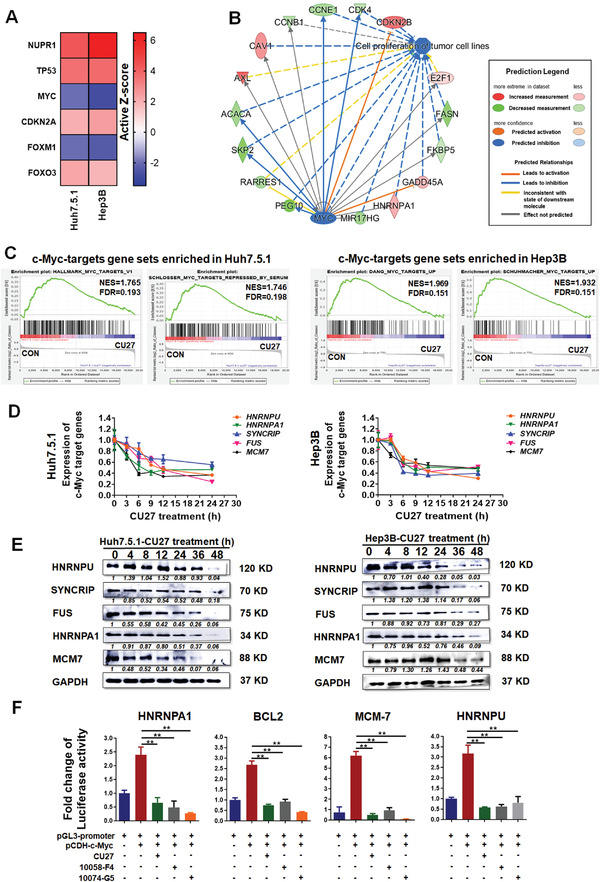
CU27 inhibits the transcriptional activity of c‐Myc in HCC cells. A) Top 6 candidate CU27 transcription factor targets shared in Huh7.5.1 and Hep3B cells, predicted by IPA. Red squares, activated transcription factors; blue squares, inhibited transcription factors. The genes are arranged by the Active Z‐score. B) Downstream biological effects of c‐Myc, predicted by IPA from the cDNA microarray. C) GSEA shows MYC‐targets related gene sets enriched in control versus CU27‐treated HCC cells (the NES absolute value ≥ 1.0, FDR not greater than 0.25, were statistically significant). D) qRT‐PCR and E) WB analysis of selected c‐Myc targets in CU27‐treated cells. Relative value = each protein band's grayscale value/GAPDH band's grayscale value. Normalized relative value = each protein band's relative value in different group/each protein band's relative value in control group. Each protein's normalized relative value was indicated under the protein band. The normalized relative value for each protein in control group is 1. F) Dual‐luciferase reporter assay. Each group was normalized to pGL3‐promoter‐transfected‐alone group (*n* = 3). Means ± SD; ^**^ *p* < 0.01; Student's *t*‐test.

Having identified the inhibitory effect of CU27 on liver CSCs, we next aimed to determine the underlying mechanisms of CU27 action. First, we performed cDNA microarray in CU27‐treated HCC cells (Figure S7A,B, Supporting Information), and confirmed the expression levels of some of these differentially expressed genes by qRT‐PCR (Figure S7C, Supporting Information) and WB (Figure S7D, Supporting Information). Next, we sought to clarify key biological targets of CU27. To address this question, we performed a classical ingenuity pathway analysis (IPA) for differentially expressed genes from the microarray. IPA showed a series of activated and suppressed transcription factors shared between two CU27‐treated HCC cells (Table S2, Supporting Information). According to the absolute value of activation Z‐score, we focused on top six transcription factors including CU27‐activated transcription factors (red graph): nuclear protein 1 (Nupr1), p53, CDKN2A‐encoded tumor suppressor p14^ARF^, and the forkhead box O3 (FoxO3); CU27‐inhibited transcription factors (blue graph): c‐Myc, the forkhead box M1 (FoxM1) (**Figure** **4**A). Among these ones, it is reported that p53,^[^ ^30^, ^31^ ^]^ CDKN2A‐encoded tumor suppressor p14^ARF^ in human,^[^ ^31^ ^]^ the forkhead box O3 (FoxO3)^[^ ^32^ ^]^ are negatively regulated in cancers by c‐Myc, a well‐known oncogenic transcription factors in CSCs;^[^ ^33^ ^]^ and the forkhead box M1 (FoxM1) is negatively by p53.^[^ ^34^ ^]^ These pieces of molecular regulation evidence indicated that c‐Myc might be a central hub regulator in CU27‐triggered change of gene profiling. A high consistency between the c‐Myc‐regulated biological effectors (Figure 4B) and the stemness‐inhibitory effects of CU27 was identified in our results. Furthermore, we moved on to perform the gene set enrichment analysis (GSEA) and found four Myc‐target gene signature sets were positively enriched in untreated cells but were negatively related to CU27‐treated cells (Figure 4C). These data promoted us to consider c‐Myc transcriptional activity might play a key role in the cancer‐specific action of CU27 in HCC. To confirm this, we first examined expressions of several typical c‐Myc‐targets after CU27‐treatment at mRNA (Figure 4D) and protein levels (Figure 4E). In dual‐luciferase reporter assay for four typical c‐Myc target genes, c‐Myc overexpression elicited a high luciferase activity, which was significantly reduced in the presence of CU27, or two well‐established c‐Myc transcription inhibitors, 10058‐F4^[^ ^35^ ^]^ and 10074‐G5,^[^ ^36^ ^]^ respectively (Figure 4F).

Together, these results show CU27 inhibits the transcriptional activity of c‐Myc in HCC cells.

### 2.4 CU27 Binds c‐Myc bHLH/LZ Domains, Inhibits c‐Myc‐Max Complex Formation, and Prevents Its Occupancy on Target Gene Promoters

**Figure 5:**
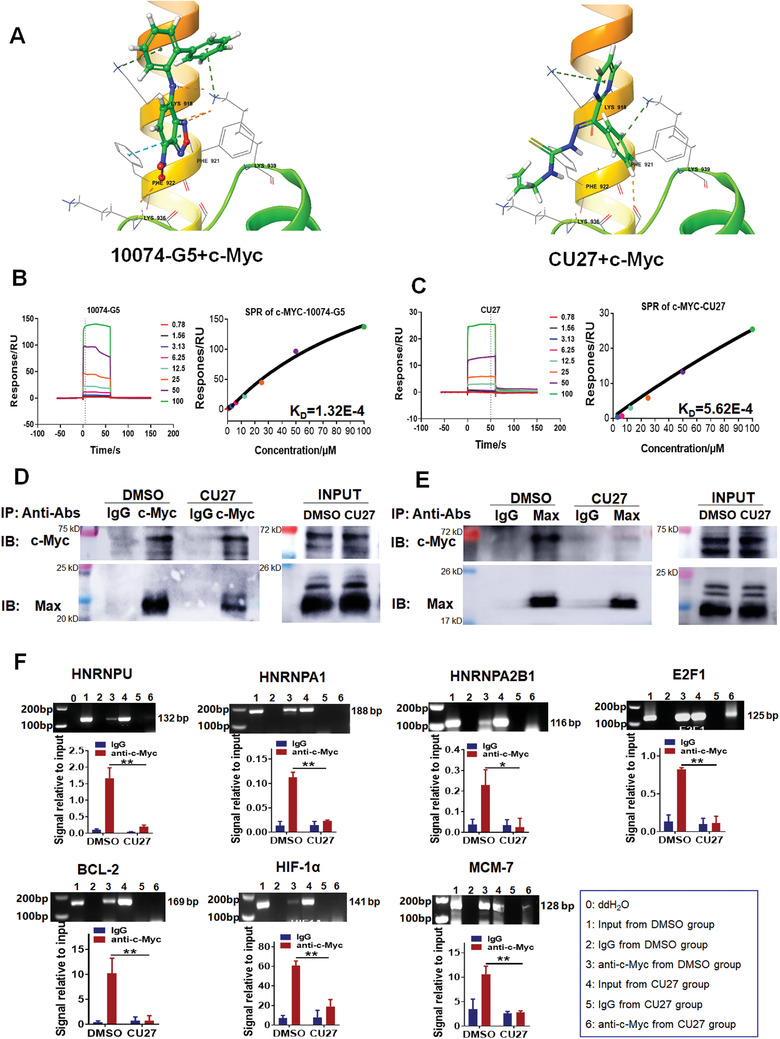
CU27 binds c‐Myc, inhibits c‐Myc‐Max complex formation, and prevents its occupancy on target gene promoters. A) Molecular docking of compounds to c‐Myc protein (ASN900‐LEU981). Graphical representation for the binding mode of 10074‐G5 or CU27 to the binding site of c‐Myc. B,C) SPR assay. Double reference subtracted sensograms of CU27 or 10074‐G5 binding to the truncated c‐Myc protein (left). The combining isotherm fitting curves for the interaction between CU27/10074‐G5 and the truncated c‐Myc protein to determine the equilibrium binding constant (KD) (right). D,E) IP analysis. Huh7.5.1 cells were exposed for 8 h of CU27 (10 µm). Total cell lysates were prepared and c‐Myc proteins (D) or Max proteins (E) were immunoprecipitated (IP) followed by immunoblotting (IB) of the precipitate with each antibody. F) ChIP analysis in Huh7.5.1 cells 24 h after treatment with CU27 (*n* = 3). Means ± SD; ^*^ *p* < 0.05; ^**^ *p* < 0.01; Student's *t*‐test.

To further clarify the mechanism of CU27 inhibiting c‐Myc transcriptional activity, we performed molecular docking of CU27 in c‐Myc (Asn900‐Leu981, PDB code 1NKP) to predict whether CU27 could interact directly with c‐Myc protein. 10074‐G5, which has been reported to directly bind bHLH/LZ domains of c‐Myc protein,^[^ ^19^, ^36^ ^]^ was set as the positive control. The results indicated CU27 exhibits a better or comparable docking ability than other agents in our leading pool including 10074‐G5, in both non‐solvent model and two solvent models (**Figure** **5**A; Table S3, Supporting Information). We then performed a surface plasmon resonance (SPR) assay, to confirm and quantify the binding between CU27 and c‐Myc protein. The results showed both CU27 and 10074‐G5 can interact with the truncated c‐Myc protein (a.a 354–437) consisting of the bHLH/LZ domain.^[^ ^37^ ^]^ Specifically, the equilibrium binding constant (*K*_D_) values of CU27 and 10074‐G5 were 5.62E‐4 µm and 1.32E‐4 µm, respectively (Figure 5B,C), suggesting CU27 binds the truncated c‐Myc protein with a comparable affinity as 10074‐G5.

Since c‐Myc‐Max complexes are known playing vital roles in transcriptional regulation of c‐Myc and its target genes,^[^ ^37^ ^]^ we conducted immunoprecipitation (IP) and chromatin immunoprecipitation (ChIP) assays to assess effects of CU27 on c‐Myc‐Max complex formation and its occupancy on target gene promoters. IP analyses suggested CU27 significantly inhibits the formation of c‐Myc‐Max complex regards of IP by anti‐c‐Myc (Figure 5D) or anti‐Max antibody (Figure 5E). Consistently, ChIP analysis showed the amount of c‐Myc target gene promoters was significantly lower in CU27‐treated cells than controls (Figure 5F).

Taken together, these results suggest CU27 binds c‐Myc bHLH/LZ domains, inhibits c‐Myc‐Max complex formation, and prevents its occupancy on target gene promoters in HCC cells.

### 2.5 CU27 is More Effective on CSC Inhibition Than 10058‐F4 and 10074‐G5

**Figure 6:**
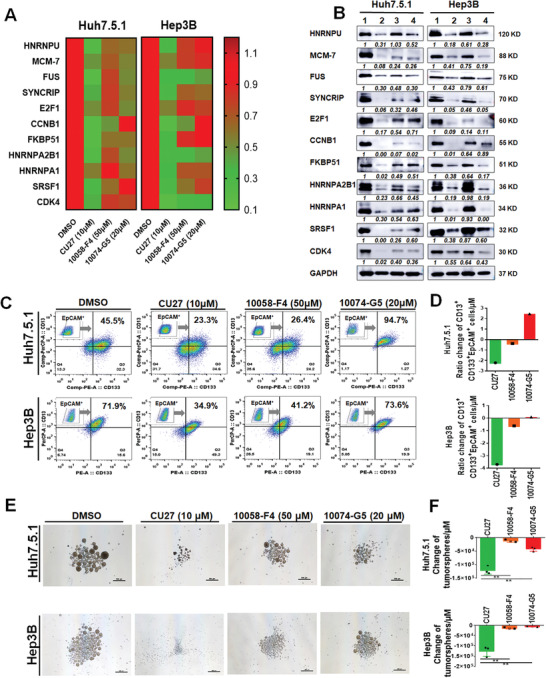
Comparison analysis of biological effects on liver CSC between CU27 and two available commercial c‐Myc inhibitors. A) qRT‐PCR analysis of selected c‐Myc target genes in HCC cells after 24 h‐treatment at the indicated concentration, shown in heat map. The DMSO group was normalized as 1. B) WB analysis of typical c‐Myc targets in HCC cells after 48 h‐treatment. Relative value = each protein band's grayscale value/GAPDH band's grayscale value. Normalized relative value = each protein band's relative value in different group/each protein band's relative value in control group. Each protein's normalized relative value was indicated under the protein band. The normalized relative value for each protein in control group is 1. Lane 1, DMSO; Lane 2, CU27 (10 µm); Lane 3, 10058‐F4 (50 µm); Lane 4, 10074‐G5 (20 µm). C) FCM analysis of the CD13^+^/CD133^+^/EpCAM^+^ CSC population in HCC cells treated with compounds. D) Normalization of the percentage change of CD13^+^/CD133^+^/EpCAM^+^ CSC population induced by per micromole of each compound. E) Representative images of tumorspheres derived from HCC cells received a 48 h‐pretreatment with each compound at indicated concentration. Scale bars, 500 µm. F) Quantitative analysis of the potency of tumorsphere inhibition for each compound, calculated by per micromole of each compound (*n* = 3), Means ± SD; ^*^ *p* < 0.05, ^**^ *p* < 0.01; Student's *t*‐test.

Next, we compared the biological effects of CU27 on CSCs with 10058‐F4 and 10074‐G5. The inhibitory potential of CU27 on c‐Myc targets were stronger than 10074‐G5 and 10058‐F4, at both mRNA (**Figure** **6**A) and protein levels (Figure 6B). Both CU27 and 10058‐F4 treatment significantly decreased the combination of three cell surface marker protein expressions of CSC population (CD13^+^/CD133^+^/EpCAM^+^), while 10074‐G5 had no inhibitory effects on CSC population in two tested cell lines (Figure 6C). The inhibitory effect of CU27 was stronger than 10058‐F4 after dose normalization (Figure 6D). For tumorsphere formation, CU27 showed higher sphere‐forming inhibitory capacity than 10058‐F4 and 10074‐G5 after dose normalization (Figure 6E,F). These data suggest CU27 is more effective on CSC inhibition than 10058‐F4 and 10074‐G5.

### 2.6 CU27 Synergizes with Sorafenib, Decreases the Primary Tumor Size and Its Metastatic Capacity in Mouse Model

**Figure 7:**
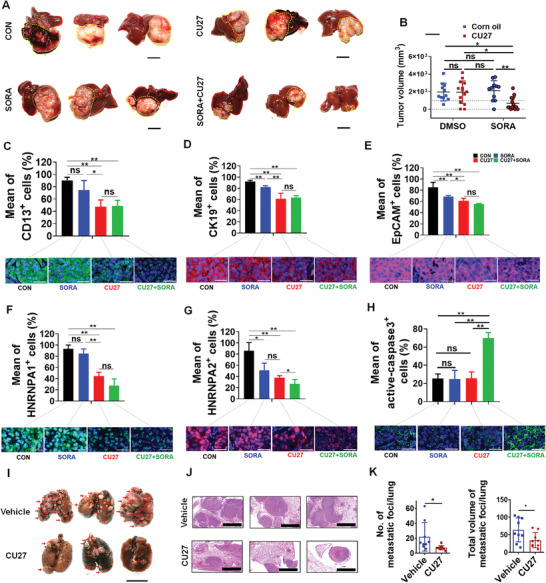
Therapeutic potential of CU27 in liver orthotopic tumor‐bearing mouse model. A) Representative liver images of intrahepatic xenografted mice in the control group (*n* = 11), the sorafenib‐monotherapy‐group (*n* = 11), the CU27‐monotherapy‐group (*n* = 14), and the combination‐treatment group (*n* = 14). Bars, 1 cm. B) Statistics of tumor volume between each group. Means ± SD; ^*^ *p* < 0.05, ^**^ *p* < 0.01; two‐way ANOVA. Representative immunohistochemistry (IHC) staining of C) CD13, D) CK19, E) EpCAM, F) HNRNPA1, G) HNRNPA2 and H) active‐caspase 3 in xenograft tumor tissues (bottom) and the statistics histogram of mean percentage (top). Bars, 30 µm. Means ± SD; ^*^ *p* < 0.05, ^**^ *p* < 0.01; Student's *t*‐test. I) Representative lung images of xenografted mice treated with CU27 or vehicle (corn oil). Red arrows, metastatic lung nodules. Bars, 1 cm. J) Representative H&E staining images of metastatic lung foci. Bars, 2000 µm. K) Statistical histogram of the number (left) and total volume (right) of metastatic lung foci. CU27 group (*n* = 38), vehicle group (*n* = 37). Six sections per lung were randomly chosen and analyzed in each group. Means ± SD; ^*^ *p* < 0.05; Student's *t*‐test, excluding the maximum and minimum values for each group.

Now that we have proved CU27 can sensitize Dox, 5‐FU, and sorafenib in HCC cells, we further aim to test the synergistic therapeutic effect of CU27 and sorafenib in a metastatic mouse model using HCCLM3 cells, which are characterized by a high c‐Myc expression level and a high metastatic potential, as we previously reported.^[^ ^26^, ^38^, ^39^ ^]^ Visible tumor nodules appeared in all the groups (**Figure** **7**A; Figure S8, Supporting Information). No difference of the liver tumor volume between sorafenib‐monotherapy‐group and control group was observed, as expected. So did the CU27‐monotherapy‐group. However, the combination‐treatment‐group showed a significant reduced tumor volume (Figure 7A,B); To the end of the 8‐week treatment, the combined therapies presented a prolonged survival trend than other groups (Figure S9, Supporting Information). A significant decreasing of CSC marker protein, including CD13^+^ population (Figure 7C; Figure S10A, Supporting Information), CK19^+^ population (Figure 7D; Figure S10B, Supporting Information), EpCAM^+^ population (Figure 7E; Figure S10C, Supporting Information), was observed in both CU27‐treated and combination‐treatment‐group compared with control group, while no difference was observed between CU27 monotherapy and combination therapy group (Figure 7C–E). Two target genes of c‐Myc, Heterogeneous nuclear ribonucleoprotein (HNRNP) A1, and HNRNPA2, were significantly downregulated in CU27 monotherapy and combination therapy group (Figure 7F,G; Figure S10D,E, Supporting Information). A strong increasing of active‐caspase3 expression was only observed in combination‐treatment‐group (Figure 7H; Figure S10F, Supporting Information). Considering that the apoptosis of seeded HCC cells in vitro or in vivo can affect the assessment of invasion and metastasis, while CU27 itself does not act by promoting cell apoptosis (Figure S4, Supporting Information), therefore, we focused on whether CU27 itself can inhibit HCC invasion and metastasis in an apoptosis‐independent manner. A significant decrease of cell invasion ability was observed in CU27‐pretreated HCCLM3 cells (Figure S11, Supporting Information). Moreover, both the number and the size of micrometastatic foci in CU27‐treated group were significantly reduced compared with control group (Figure 7J,K; Figure S12, Supporting Information). In addition, no significant lesions were identified after pathological analysis of the main organs in CU27‐treated mice (Figure S13, Supporting Information).

These findings demonstrated CU27 can synergize with sorafenib, decrease the primary tumor size and its metastatic capacity in vivo.

## 3 Discussion

Most of the previous studies focused on the cancer chemoprevention role of selenium as dietary supplementation, which is based on the findings that low selenium levels are associated with an increased cancer risk,^[^ ^40^, ^41^, ^42^, ^43^ ^]^ especially for the liver.^[^ ^44^ ^]^ However, whether CSC might represent a real target cell population of selenium compound is not thoroughly addressed in liver cancer. In this study, we provide evidence that a new selenium‐containing compound CU27, identified from our designed focus library, inhibits CSC, resulting in metastasis attenuation and sorafenib sensitization in HCC.

Interestingly, selenium compounds are characterized by specific enrichment in cancer cells. As early as the 1960s, radioactive selenite (^75^SeO_3_^2−^) was used at low doses (approximately nm levels in plasma) as a tumor‐localizing agent by intravenous injection to detect malignant tissues in the brain and thoracic cavity of patients, although the cancer‐specific cytotoxic effects of selenite were not known at the time.^[^ ^45^, ^46^ ^]^ But inorganic selenium compounds have poor lipid solubility, are difficult to penetrate through the cell membrane, are easy to accumulate in the body, and have large side effects, so it is difficult to be used directly as an anti‐cancer drug. Therefore, efforts have been made to design and develop a number of novel organic selenium molecules,^[^ ^23^ ^]^ because they have a higher antitumor activity, lower toxicity, and good biocompatibility than inorganic selenium compounds. Our designed organic selenium compound CU27, belonging to one of selenourea molecules with more anti‐tumor activity compared with the corresponding sulfur analogues.^[^ ^47^ ^]^ Here, we found CU27 can exert anti‐HCC effects by inhibiting the action of CSC, possibly by promoting HCC differentiation.

**Figure 8:**
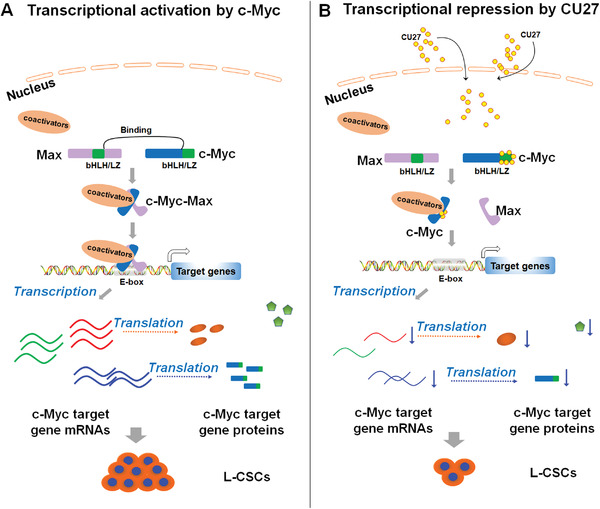
Working hypothesis for the mechanism of CU27 action in liver CSC (L‐CSCs). A) The dimerization of Myc with Max by direct interaction of the basic‐helix‐loop‐helix/leucine zipper (bHLH/LZ) domains makes them cooperative binding to E‐box (CACGTG or CATGTTG)‐contained promoters of c‐Myc and its target genes (HNRNPU, MCM7, E2F1, HNRNPA1, etc.). This process leads activation of expressions of c‐Myc and its target genes, which promote self‐renewal of CSCs. B) CU27 can bind c‐Myc bHLH/LZ domain, promote c‐Myc‐Max complex dissociation, and prevent its occupancy on target gene promoters, inhibited the expression of c‐Myc and its target genes. This effect leads to the suppression of L‐CSC self‐renewal and CSC‐associated traits (such as chemoresistance and metastasis).

For unrevealing molecular mechanisms of CU27 inhibiting liver CSC, we first obtained differential gene expression profiles of CU27‐treated HCC cells, followed by integrating our IPA data and published literatures, leading us to hypothesize c‐Myc transcriptional activity may be the major target of CU27. The hypothesis is further confirmed by series of assays including GSEA, dual‐luciferase reporter assays, expression levels of typical c‐Myc targets, molecular docking, SPR, IP, and ChIP in our study. Besides current underlying mechanism we have proposed (**Figure** **8**), we also note there may be an alternative mechanism that CU27 dually targets tumor suppressor p53 (or p53‐Nupr1complex^[^ ^48^ ^]^) and c‐Myc, as well as Sheela A Abraham et al reported in leukemia stem cells.^[^ ^49^ ^]^ This may lead to synergistic effects of cell target, differentiation, and elimination of CSC at molecular level.

The commercially available inhibitors 10058‐F4 and 10074‐G5 have been widely used in specifically inhibiting the c‐Myc transcription activity and the growth of c‐Myc‐expressing cells,^[^ ^19^ ^]^ but they are reported to be lack of significant antitumor activity in vivo due to their rapid metabolisms and low concentrations in tumors,^[^ ^35^, ^36^ ^]^ leading to the limitations of their clinical applicability. We compared and discovered that CU27 has no significant differences with 10058‐F4 and 10074‐G5 in terms of binding site, binding capacity, and transcription‐regressed function for c‐Myc, while it can more effectively target CSC than them in tumorsphere assay, even at a lower concentration. Besides, we have provided pieces of evidence in mouse model that CU27 has the capacity to decrease the CSC frequencies and inhibit distant metastasis. To our knowledge, it is the first time to synthesize a selenium‐containing small molecule with CSC inhibition potential, at least partially through blocking c‐Myc transcription activity.

Under the effective dose and duration of CU27 treatment in HCC, cell apoptosis is not significantly induced. This is different from Quarfloxin (CX‐3453), a specifically designed inhibitor to induce apoptosis by reducing c‐Myc mRNA in neuroendocrine carcinoma.^[^ ^50^ ^]^ However, we observed that CU27 may sensitize cancer cells to chemotherapeutics, especially current first‐class drug sorafenib in vitro and in vivo. IHC results of mice‐bearing tumor showed that sorafenib can downregulate CK19^+^, EpCAM^+^ cell population but not CD13^+^ cell. Compared with the sorafenib group, CU27 could further downregulate CD13^+^, CK19^+^, and EpCAM^+^ cell population, respectively. Compared with the CU27 group, the combination therapy group had no significant downregulation of CD13^+^, CK19^+^, and EpCAM^+^ population, respectively. These results indicated there is no synergistic effect between CU27 and sorafenib in the downregulation of CSC population, which is mainly mediated by CU27. For two canonical target genes of c‐Myc, HNRNPA1 and HNRNPA2, as well as CSC marker proteins, there is no synergistic effect of CU27 and sorafenib in the downregulation of the proportion of HNRNPA1^+^ cells, which is mainly mediated by CU27. However, combination therapy group further decreased the proportion of HNRNPA2^+^ cells compared with the CU27 group, indicating a synergistic effect in the downregulation of the proportion of HNRNPA2^+^ cells between CU27 and sorafenib. Given that c‐Myc has been reported to regulate hundreds of target genes directly or indirectly,^[^ ^33^ ^]^ this also reflects the complexity of the regulation of c‐Myc target genes by these two drugs in vivo. For active‐Caspase 3, compared with the control group, there was no difference between the sorafenib and CU27 groups, while the combination therapy group significantly increased active‐Caspase 3^+^ cells, indicating a significant synergistic effect of CU27 and sorafenib in HCC cell apoptosis. This is consistent with our observation of significantly reduced tumor volumes in the combination treatment group (Figure 7A,B). Taken together, these in vivo IHC results further support our hypothesis that CU27 promotes the sensitivity of sorafenib‐resistant HCC cells to sorafenib by downregulating the CSC proportion. These data supported the notion that combination therapies, which target both the CSC population and the bulk of the cancer cells, will be required to enhance anti‐cancer effects of CU27 for success in clinical and therapeutic implications.

## 4 Conclusion

Collectively, we have developed a novel organic selenium compound, CU27, which can bind c‐Myc bHLH/LZ domains, block c‐Myc‐Max complex formation, and prevent its occupancy on target gene promoters in HCC cells. These effects lead to the suppression of CSC self‐renewal (Figure 8), resulting in inhibition of metastasis and sorafenib resistance in HCC, which sheds light on the therapeutic potential of CU27 for HCC patients.

## 5 Experimental Section

### General Synthesis Procedures for Organic Selenium/Sulfur Compounds

1) Chemistry: In a general procedure (Figure S1, Supporting Information), the condensation reaction occurred between the substituted amino compounds, carbon disulfide and triethylamine. Then the thiosemicarbazones (compound 1) were created followed by the hydrazine substitution reaction. The thiosemicarbazones (compound 1) were converted to the S‐substituted thiosemicarbazones (compound 2) by reaction with methyl iodide, followed by the substitution reaction by NaHSe, the amino selenoureas (compound 3) were obtained in high yields. Compound 3 reacted with disubstituted ketones in acetic acid to yield the compound 4. Compound 2 reacted with disubstituted ketones in acetic acid to yield the compound 5. 2) Experimental: 1H NMR and 13C NMR spectra were recorded at 400 and 100 MHz on JNM‐ECA‐400 instrument in the solvent indicated below, respectively. Proton and carbon chemical shifts are expressed in ppm relative to internal tetramethylsilane and coupling constants (*J*) are expressed in Hertz. Melting points were determined using a RY‐1 apparatus. Thin‐layer chromatography was carried out on silica gel GF/UV 254 and the chromatograms were performed on silica gel (200–300 mesh) visualized under UV light at 254 and 365 nm. 3) General procedures for 4: Thiosemicarbazones (compound 1) (10 mmol) and methyl iodide (10 mmol) were added in absolute methanol (30 mL), and the solution was stirred for 1h at reflux temperature. The reaction mixture was cooled to room temperature and concentrated. The S‐substituted thiosemicarbazones (compound 2) was obtained as a solid; yield: about 90%. NaBH_4_ (13 mmol) and selenium powder (10 mmol) were added in absolute methanol (10 mL) at 0 °C under N_2_, and it was stirred for 40 min. Sodium carbonate (8 mmol) was added. And then compound 2 (8 mmol) was added into the NaSeH solution and the reaction mixture was stirred 20 h at room temperature. Acetic acid was added dropwise into the reaction mixture within 30–60 min. Meanwhile, the nitrogen was passed and the exhaust was absorbed by 5% lead acetate solutions. The compound 3 was obtained by silica gel chromatography as a white solid in 50–60% yield. The amino selenoureas (compounds 3, 3 mmol) and disubstituted ketones (3 mmol) were added in absolute methanol (30 mL), followed by 5 drops of acetic acid added. It was stirred for 4 h at reflux temperature. The reaction solution was cooled and the compound 4 were obtained by filtered as a yellow solid in 50–70% yield. 4) Phenyl‐2‐pyrimidinyl ketone 4‐allyl‐3‐amino selenourea (4a, CU27): ^1^H‐NMR (400 MHz, DMSO) *δ*ppm: 4.33–4.36 (m, 2H), 5.12–5.15 (m, 2H), 5.90–5.94 (m,1H), 7.40–7.42 (m, 3H), 7.67–7.69 (m, 3H), 9.08–9.09 (d, 2H), 9.40–9.41 (m, 1H), 12.94 (s, 1H.). HLPC‐MS m/z:346.1 [M+1]^+^.

### Cell Culture

HCC cell lines (Huh7.5.1, Hep3B, HCCLM3, and PLC/PRF/5) and the L02 human fetal liver cell line were used as described previously.^[^ ^26^, ^51^ ^]^ Huh7.5.1 cells were maintained in our laboratory, as described previously. Hep3B cells were purchased from National Infrastructure of Cell Line Resource (Beijing, China). HCCLM3 cells were obtained from the Liver Cancer Institute of Fudan University (Shanghai, China). The PLC/PRF/5 was obtained from ATCC. The HEK‐293T packaging cell line was acquired from Invitrogen (Carlsbad, CA). All cells were maintained in Dulbecco's modified Eagle's medium (Sigma) containing 10% fetal bovine serum (ExCell Biology, Shanghai, China), at 37 °C and 5% CO_2_, except for Hep3B (in MEM, Sigma) and L02 (in RPMI 1640 medium, Sigma). All reagents used in this study are summarized in Table S4, Supporting Information.

### Small Molecule Compounds Screening by Cell Viability Assay

The L02 and Hep3B cells were seeded in 96‐well plates (5000 cells per well), 24 h later, the cells were treated with DMSO (Control group) or each small molecule compound (10 µm) for another 48 h. Then cells were treated with CCK‐8 reagent (Dojindo Laboratories, Kumamoto, Japan) and incubated at 37 °C for 2 h. Absorbance at 450 nm was measured by SpectraMax M2e (Molecular devices, San Jose, CA). Five replicate wells for each group and three independent experiments were conducted. The cell viability (%) of each small molecule compound was normalized as the ratio of each compound/DMSO in L02 and Hep3B cells, respectively. The relative cell viability (%) = The cell viability of L02 cells (%) − The cell viability of Hep3B cells (%).

### Small Molecule Compounds Screening by 3D Tumor Spheroid Assay

The 3D tumor spheroids were generated using the U‐shaped‐bottom 96‐well plate (#262162, Thermo Fisher Scientific, Waltham, MA). Tumor cells were seeded at the density of 5000 cells per well onto the microwells and were spun down at 1200 rpm for 5 min. The same culture medium for tumorsphere formation was used. The test compounds were added at day 4 when tumor cells aggregate and spheroids formed.

### Small Molecule Compounds Screening by Tumorsphere Formation Assay

HCC cells were received a 48‐h pre‐treatment with candidate test compound (10 µm) before tumorsphere culture. For tumorsphere formation assay, HCC cells (2000 cells per well) were seeded in ultra‐low attachment six‐well plates (Corning, Corning, NY) in serum‐free DMEM/F‐12 medium (GIBCO, Grand Island, NY); the serum‐free DMEM/F‐12 medium contained N2 (1:100, GIBCO), B27 (1:50, GIBCO), EGF (10 ng mL^−1^; R&D, Minneapolis, MN), bFGF (10 ng mL^−1^; PeproTech, Rocky Hill, NJ), penicillin G (100 µg mL^−1^), streptomycin (100 U mL^−1^), and insulin (4 mg mL^−1^; all from Sigma, St. Louis, MO). After 1–2 weeks, bright field images of tumorspheres were captured and quantitatively analyzed with Image‐Pro Plus 6.0 software (Media Cybernetics, Inc., Maryland). The results from three replicate wells in each group and at least three independent experiments were analyzed.

### Flow Cytometry and Cell Sorting of Liver CSCs

Cells were analyzed or sorted on a FACS Aria II cell sorter (BD Bioscience, San Jose, CA), as previously described.^[^ ^26^, ^52^ ^]^ For cell surface marker analysis, cells were trypsinized and washed twice with PBS. Then, 50 *μ*L diluted antibodies (PerCP‐Cy5.5‐conjugated anti‐human CD13, APC‐conjugated anti‐human EpCAM, and PE‐conjugated anti‐human CD133) were added to the cell suspensions and incubated at room temperature for 30 min. After incubation, the cells were washed three times with PBS before analysis or sorting on a FACS Aria II cell sorter (BD Bioscience, San Jose, CA). The purity of the sorted CD13^+^/EpCAM^+^/CD133^+^ population was verified by FCM. The enriched, triple‐positive HCC cells were maintained in chemically defined medium, consisting of a 1:1 mixture of DMEM/F‐12 and neurobasal medium, 0.5 × N2, 0.5 × B27, 0.1% BSA, penicillin G (100 µg mL^−1^), streptomycin (100 U mL^−1^), 2 mm L‐glutamine, 0.1 mm 2‐mercaptoethanol, 10^−7^ mol L^−1^ dexamethasone, 20 ng mL^−1^ TGF‐*α*, 10 ng mL^−1^ BMP4, 10 ng mL^−1^ bFGF, 10 ng mL^−1^ EGF, and 20 ng mL^−1^ HGF. For intracellular FCM analysis, cells were digested and fixed with 4% paraformaldehyde for 15 min, followed by 0.2%Triton‐x‐100 membrane rupturing for 15 min. The cells were washed once with PBS and blocked with 10% goat serum for 30 min. Then discarded the supernatant by centrifugation and incubated with primary antibody (1:400 dilution in PBS) and incubate overnight. After washed three times with PBS, the cells were incubated with the secondary antibody at room temperature for 60 min. Washed three times with PBS before analysis. Isotype indicated the cells incubated with only the secondary fluorescent antibody without the primary antibody. The antibodies used are in Table S5, Supporting Information.

### Immunofluorescence Staining

Immunofluorescence staining was performed as previously described^[^ ^51^ ^]^ In brief, fixed cells were blocked and incubated with primary antibodies overnight at 4 °C, followed by incubation with fluorescence‐conjugated secondary antibodies, according to the manufacturer's instructions. The nuclei were stained with DAPI. Fluorescence images were captured with an LSM880 confocal system (Zeiss, Carl Zeiss MicroImaging, Jena, Germany). For multiplexed immunohistochemistry, it was performed with PANO 7‐plex IHC kit (#0004100100, Panovue, Beijing, China).^[^ ^53^ ^]^ Tissue section image was acquired with TissueFAXS (TissueGnostics GmbH, Vienna, Austria) with a Zeiss Axio Imager Z2 Microscope System, and quantitatively analyzed by HistoQuest/TissueQuest software (version 7.0.1, Vienna, Austria).

### Cell Viability and Chemoresistance Assays

A CCK‐8 kit was used to determine cell viability, as previously described.^[^ ^26^ ^]^ Experiments were conducted in triplicate with five replicate wells for each analysis. A total of 2 × 10^3^–3 × 10^3^ cells per well were seeded in 96‐well plates. The medium was then replaced with CCK‐8 working solution and incubated at 37 °C for 2 h. The absorbance at 450 nm was measured on a SpectraMax M2e (Molecular Devices, Sunnyvale, CA). For the doxorubicin (Sangon Biotech, Shanghai, China)/sorafenib (Santa Cruz, Santa Cruz, CA)/5‐FU (Sangon Biotech, Shanghai, China) chemoresistance assay, 4 × 10^3^–5 × 10^3^ cells per well were seeded in 96‐well plates. After 24 h, the medium was replaced with medium containing the indicated concentrations of the chemotherapeutic agents with or without CU27. The absorbance at 450 nm was measured at the indicated time point.

### Microarray Analysis and Data Mining

The microarray experiments were performed based on the standard protocols (OE Biotech Company, Shanghai, China). In brief, total RNA was transcribed and labeled with biotin before the labeled RNAs were hybridized onto the microarray. After washing, the arrays were scanned by an Affymetrix Scanner 3000 (Affymetrix, USA). The array images were analyzed with an Affymetrix GeneChip Command Console (version 4.0, Affymetrix), followed by basic analysis with GeneSpring software (version 13.1; Agilent Technologies). The raw data were normalized via the RMA algorithm. Differential expression was identified based on the fold change (FC) and *p* value. The threshold established for differential expression was FC > 1.5 and *p* <0.05. The functional annotation of the differentially expressed genes was performed by OE Biotech Company (Shanghai, China) and included GO analysis, Kyoto Encyclopedia of Genes and Genomes analysis, and pathway network analysis. Finally, differentially expressed genes in the microarray analysis were subjected to IPA to predict upstream regulators.

### Reverse Transcription PCR and Quantitative Real‐Time PCR

Total RNA was isolated using TRIzol reagent (Invitrogen, Carlsbad, CA). Reverse transcription and RT‐qPCR were performed with ReverTra Ace qPCR RT Master Mix (TOYOBO, Shanghai) and THUNDERBIRD SYBR qPCR Mix (TOYOBO), as previously described.^[^ ^26^ ^]^ Gene expression was quantified based on the ΔΔC_t_ method and normalized to the expression of the reference gene, GAPDH. All primers used in this study are summarized in Table S6, Supporting Information.

### Western Blot

Western blot was carried out as previously described.^[^ ^26^ ^]^ Briefly, cell pellets were collected and lysed in RIPA lysis buffer (CWBIO, Beijing, China) containing protease inhibitors (Roche, Mannheim, Germany). The immunoreactivity on transferred PVDF membranes was detected with Immobilon Western Chemiluminescent HRP Substrate (Merck Millipore). All antibodies used in this study are summarized in Table S5, Supporting Information.

### Chromatin Immunoprecipitation

ChIP was performed using an EZ‐ChIP Chromatin Immunoprecipitation Kit (#17‐295, Merck Millipore, Boston, MA), according to the manufacturer's instructions. Briefly, 10^7^ cells were fixed with 1% formaldehyde at room temperature for 10 min. The fixed cells were then collected, lysed, and then sonicated by six cycles of 10 s on/20 s off in 30% AMPL with a Cole‐Parmer CPX 130 Ultrasonic Processor (Cole‐Parmer, Vernon Hills, IL). Cell lysates were pre‐cleared with protein A/G agarose beads at 4 °C for 1 h. A c‐Myc antibody (10 µg per test) or rabbit isotype control IgG (2 µg per test) was added to the precleared cell lysates and rotated overnight at 4 °C. Protein A/G agarose beads were then incubated with the protein/DNA complexes for 1 h at 4 °C with rotation. The crosslinks between the protein and DNA fragments were then reversed to free the c‐Myc‐binding DNA fragments. The purified DNA fragments were then analyzed by RT‐qPCR. Antibodies are listed in Table S5, Supporting Information. The primer sequences used in the ChIP assay are listed in Table S6, Supporting Information.

### Immunoprecipitation

IP analyses were performed with protein A/G mix magnetic beads (Merck Millipore), according to the manufacturer's instructions.^[^ ^51^ ^]^ 10^7^ Huh7.5.1 cells or CU27‐treated Huh7.5.1 cells (10 µm, 8 h) were collected and lysed in 1 mL Pierce IP lysis buffer (Thermo, USA) for 30 min on ice, followed by centrifugation at 12 000 × *g* for 15 min. 100 µL of cell lysates were used as Input. Pre‐clear the rest of cell lysates with 30 µL protein A/G mix magnetic beads (Merck Millipore) at 4 °C for 30 min with rotation. The specific antibody (anti‐c‐Myc or anti‐Max) incubated with 50 µL protein A/G mix magnetic beads at 25 °C for 60 min with rotation. The pre‐cleared cell lysates were immunoprecipitated with coated magnetic A/G mix beads and rotated overnight at 4 °C, then collected the immunocomplexes and washed three times with lysis buffer. The immunocomplexes were eluted with 50 µL SDS sample loading buffer and boiled for 5 min, then subjected to SDS‐PAGE for immunoblot analyses by antibodies (anti‐c‐Myc and anti‐Max). Antibodies are listed in Table S5, Supporting Information.

### Dual Luciferase Reporter Assay

The pCDH‐Flag‐c‐Myc plasmid (Addgene, #102626) was purchased from Addgene. The pGL3‐c‐Myc‐target‐gene‐promoter vectors were constructed by PCR amplification of the promoter regions of the c‐Myc target genes of interest from human genomic DNA and inserting PCR fragments into the pGL3‐basic vector (Promega, Madison, WI). HEK‐293T cells were seeded in 24‐well plates and divided into six groups with four‐well replicates. The assay was performed in triplicate. The cells were co‐transfected with 300 ng pCDH vectors (pCDH‐NC or pCDH‐c‐Myc) and 400 ng pGL3 vectors (pGL3‐control or pGL3‐c‐Myc). After 36 h of transfection, the cells were treated for 12 h with DMSO, 10058‐F4 (50 µm), 10074‐G5 (50 µm), or CU27 (10 µm). The pRL‐Renilla‐luciferase vector (Promega, Madison, WI) was used to normalize the differences in transfection efficiency between groups. The cells were lysed, and luciferase activity was measured using a Dual Luciferase Reporter Assay System (Promega, Madison, WI) according to the manufacturer's protocol. The primers used for PCR cloning of c‐Myc promoter are in Table S6, Supporting Information.

### Molecular Docking

The binding free energies are estimated between all of compounds (In‐House Library) and c‐Myc (PDB Code: 1NKP) using calculate binding energies protocol by Accelrys Discovery Studio V2.5. The binding free energy for a receptor‐ligand complex can be calculated from the free energies of the complex, the receptor, and the ligand.^[^ ^54^ ^]^ The optimal binding poses of compounds were selected from the docking results. All calculations for binding free energy is performed using CHARMm force field. All remaining parameters were left at their default settings. Using CHARMm‐based energies and non‐solvent model or implicit solvent model, it was possible to estimate these free energies and thus calculate an estimate for the overall binding free energy.

### Surface Plasmon Resonance Analysis

SPR analysis was performed in Pharmaron Company (Beijing, China). Briefly, human truncated c‐Myc protein (amino acids 354–437) was purified by DEAE ion exchange columns and prepared for SPR analysis. Different concentrations of 10074‐G5 (0; 0.78; 1.56; 3.12; 6.25; 12.5; 25; 50; 100 µm) and CU27 (0; 0.78; 1.56; 3.12; 6.25; 12.5; 25; 50; 100 µm) were used to interact with c‐Myc coupled to CM5 chips, and the *K*_D_ values were calculated by Biacore S200 (#29136940, GE healthcare, Shanghai, China).

### Animal Studies

NOD/SCID mice and Nu/Nu mice (male, aged 3–4 weeks) were purchased from Vital River Laboratories (Beijing, China). All mice were housed and animal studies were conducted according to protocols approved by the Institutional Animal Care and Use Committee in compliance with Beijing Medical Experimental Animal Care Commission.

For the limiting dilution assay, 1 × 10^6^, 1 × 10^5^, or 1 × 10^4^ CU27 (10 µm)/DMSO‐pretreated PLC/PRF/5 or Huh7.5.1 cells were injected subcutaneously into the left or right flanks, respectively, of NOD/SCID mice. Mice were anesthetized before injections, and the Matrigel (1:3 diluted) was added to the cell suspension solution (100 *μ*L per injection), which would be injected subcutaneously to the distal end of the mouse skin until a visible pustule appears. The hypodermic needle was held down when withdrawing the needle, pulled it out slowly, and checked to ensure that no cell suspension leaked out. After 5–6 weeks, the mice were sacrificed, and the formed tumors were examined.

For in vivo metastasis assay, a xenograft mouse model was established by intrahepatic injection of 1 × 10^6^ HCCLM3 cells into male Nu/Nu mice.^[^ ^51^ ^]^ 3 days after transplantation, the mice were randomly divided into two groups, and then underwent intraperitoneal injection of CU27 dissolved in corn oil (10 mg kg^−1^, *n* = 38) or corn oil alone (at an equal volume as the CU27 solution; *n* = 37) twice weekly. After 2 months, the mice were sacrificed, and the lungs were harvested. The lung metastatic nodules were analyzed in tissue sections after H&E staining.

For in vivo sorafenib resistance assay, intrahepatic xenograft mice were randomly divided into four groups, the control group (DMSO dissolved by corn oil, *n* = 11), the sorafenib‐monotherapy‐group (30 mg kg^−1^, *n* = 11), the CU27‐monotherapy‐group (10 mg kg^−1^, *n* = 14), and the combination treatment group (30 mg kg^−1^ sorafenib and 10 mg kg^−1^ CU27, *n* = 14). Sorafenib was dissolved by corn oil and taken by oral administration (p.o.), CU27 was dissolved by corn oil and taken by intraperitoneal injection (i.p.). The drugs were given three times per week. The mice were sacrificed after 8‐week‐treatment.

### Light Microscopy Imaging

Bright field images of cells and tumorspheres were captured under an ECLIPSE TE2000‐U microscope (Nikon Corporation Precision Equipment Company, Japan). Bright field images of HE staining were captured with a Pannoramic MIDI/P250 digital scanner (3D HISTECH, Budapest, Hungary).

### Statistical Analyses

Statistical graphs were generated and analyzed using GraphPad Prism 7 software (GraphPad Software, Inc., San Diego, CA), unless otherwise specified. The 3D‐spheres areas of Hep3B, tumorsphere areas of HCC cells, and each protein band's grayscale of western blot were analyzed with Image‐Pro Plus 6.0 software (Media Cybernetics, Inc., Maryland). Immunofluorescence staining of CD13, CK19, EpCAM, HNRNPA1, HNRNPA2, and active‐Caspase 3 were quantitatively analyzed by HistoQuest/TissueQuest software (version 7.0.1, Vienna, Austria). The statistical significance of differences between two groups was determined by a two‐tailed unpaired *t*‐test. Overall test for differences in liver CSC frequencies in in vivo limiting dilution assay were analyzed by ELDA software,^[^ ^29^ ^]^ a plot of the log proportion of nonresponding versus the number of cells, the slope of the line representing the estimated log‐liver CSC fraction and the dotted lines give the 95% confidence interval. Synergistic effects in chemoresistance assay, and differences of tumor volume between four groups (control group, the sorafenib‐monotherapy‐group, the CU27‐monotherapy‐group, and the combination‐treatment group) in intrahepatic xenografted mice model were determined by two‐way ANOVA test. All data are presented as the means ± SD. ^*^ *p* < 0.05, or ^**^ *p* < 0.01 was considered statistically significant.

## Conflict of Interest

The authors declare no conflict of interest.

## Authors Contribution

J.‐N.Z., B.Z., and H.‐Y.W. contributed equally to this work. W.Y., W. Z., X.‐T.P. supervised and coordinated all aspects of the work. J.‐N.Z., B.Z., and H.‐Y.W. performed the experiments, analyzed data, and prepared the draft manuscript. D.‐X.W. and M.‐M.Z. assisted with in vivo and in vitro experiments. M.Z., X.‐K.W., S.‐Y.F., X.‐B.Z., S.L., and W.Z. contributed in the design, synthesis of the target compound pool, and molecular docking. Q.Z. helped with molecular experiments. Y.‐C.X., contributed animal model. Y.‐L.J., J.‐F.X., X.N., and L.‐J.H provided technical or material support. J.‐N.Z., W.Y. designed the research, analyzed data and wrote the manuscript. W.Z., W.Y., X.‐T.P. conceived the project, analyzed data, and revised the manuscript.

## Supporting information

Supporting Information

## Data Availability

The data that support the findings of this study are openly available in GEO at http://www.ncbi.nlm.nih.gov/geo/, reference number 130666.
