# Supplementary material for: A Functional Screening Identifies a New Organic Selenium Compound Targeting Cancer Stem Cells: Role of c‐Myc Transcription Activity Inhibition in Liver Cancer
Source: Adv Sci (Weinh). 2022 Jun 2;9(22):2201166. doi: 10.1002/advs.202201166 (PMC9353477; doi:10.1002/advs.202201166)
Supplement: Supplementary file 1 — Supporting Information [file ADVS-9-2201166-s001.pdf]

ADVANCED SCIENCE
Open Access

Supporting Information

for *Adv. Sci.*, DOI 10.1002/advs.202201166

A Functional Screening Identifies a New Organic Selenium Compound Targeting Cancer Stem Cells: Role of c-Myc Transcription Activity Inhibition in Liver Cancer

Jun-Nian Zhou, Biao Zhang, Hai-Yang Wang, Dong-Xing Wang, Ming-Ming Zhang, Min Zhang, Xiao-Kui Wang, Shi-Yong Fan, Ying-Chen Xu, Quan Zeng, Ya-Li Jia, Jia-Fei Xi, Xue Nan, Li-Juan He, Xin-Bo Zhou, Song Li, Wu Zhong, Wen Yue* and Xue-Tao Pei**

Supporting Information

A Functional Screening Identifies a New Organic Selenium Compound Targeting Cancer Stem Cells: Role of c-Myc Transcription Activity Inhibition in Liver Cancer

Jun-Nian Zhou, Biao Zhang, Hai-Yang Wang, Dong-Xing Wang, Ming-Ming Zhang, Min Zhang, Xiao-Kui Wang, Shi-Yong Fan, Ying-Chen Xu, Quan Zeng, Ya-Li Jia, Jia-Fei Xi, Xue Nan, Li-Juan He, Xin-Bo Zhou, Song Li, Wu Zhong, Wen Yue*, Xue-Tao Pei**

This file includes:

Supplementary Figure S1-S13 with Legends

Supplementary Table S1-S6

Figure. S1.

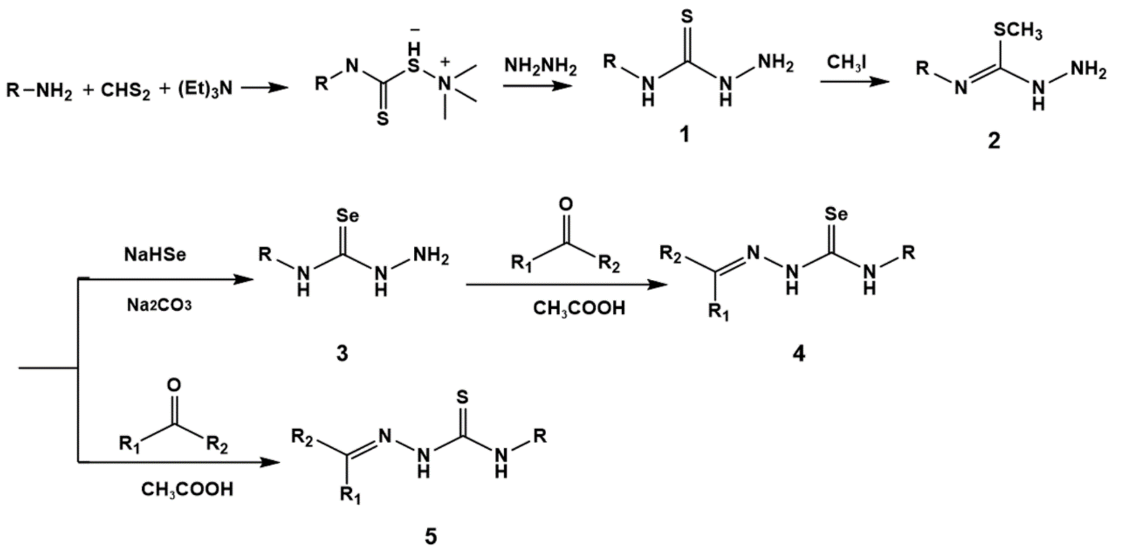

Figure S1. The synthesis process of small molecular compounds.

Figure. S2.

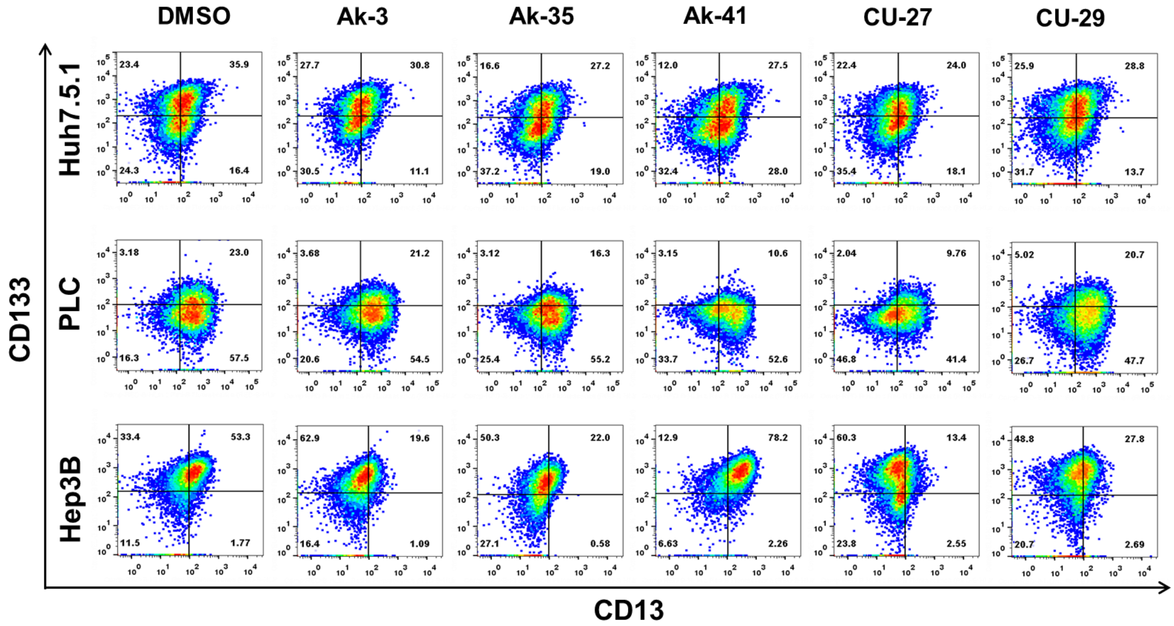

Figure S2. Flow cytometry analysis of CD13 and CD133 expression in HCC cells treated with each candidate compound. One of the flow cytometry analysis scatter plots corresponding to Fig. 1D is shown. The control is DMSO. The compound concentration is 10 μM.

Figure. S3.

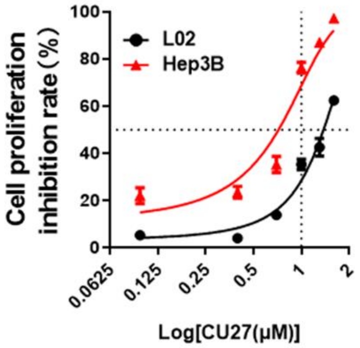

Cells	IC50 of CU27 (μM)
Hep3B	5.24±1.12
L02	23.98±1.50

Figure. S3. The proliferation dose-response curves of CU27.

Figure S4.

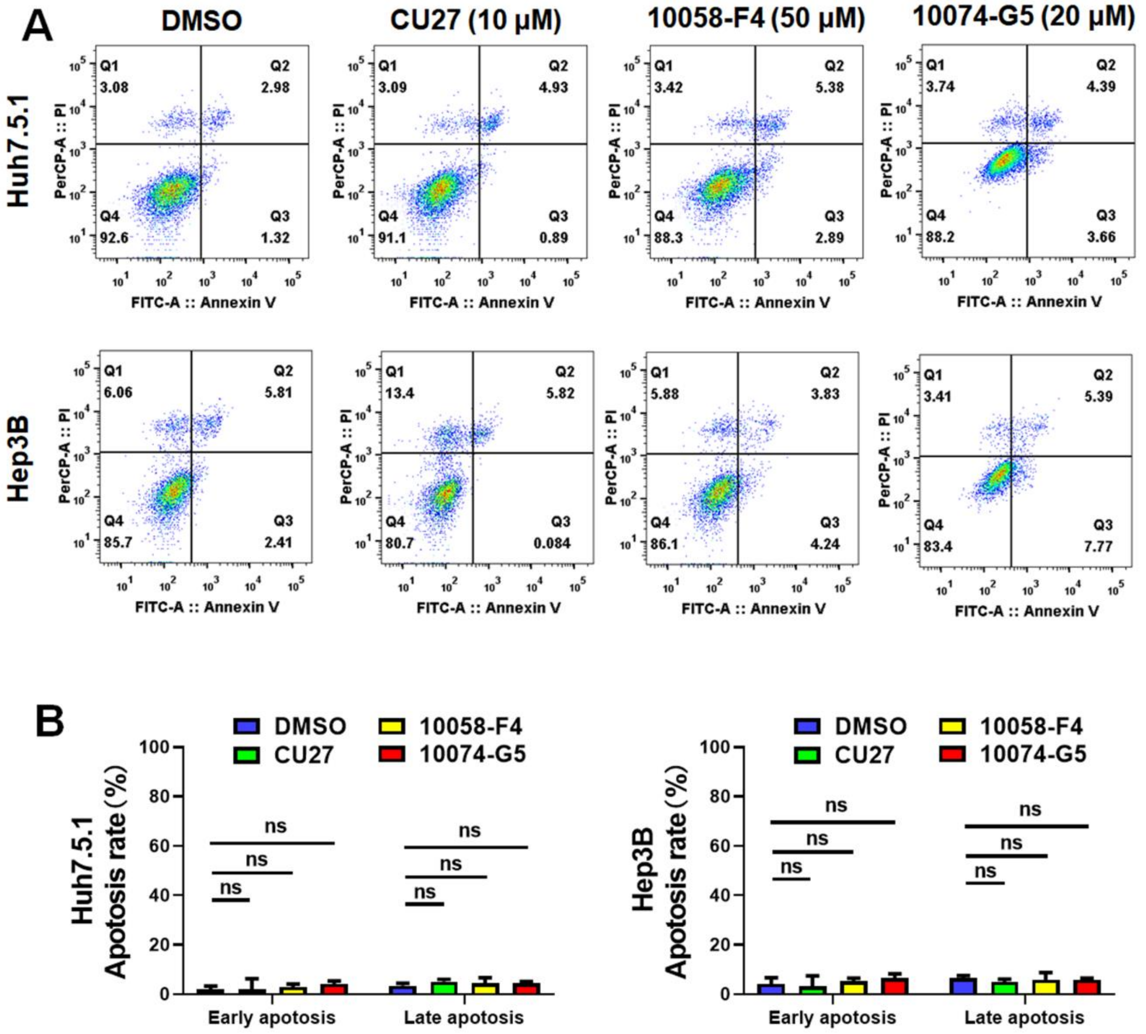

Figure S4. Cell apoptosis assay of CU27 and c-Myc inhibitors in HCC cells. Cell apoptosis was detected with Annexin V-FITC/PI by flow cytometry in HCC cells after 48-hour treatment with vehicle (DMSO), CU27, 10058-F4 and 10074-G5, respectively. Means ± SD; “ns” indicates no significant difference; Student’s t test.

Figure. S5.

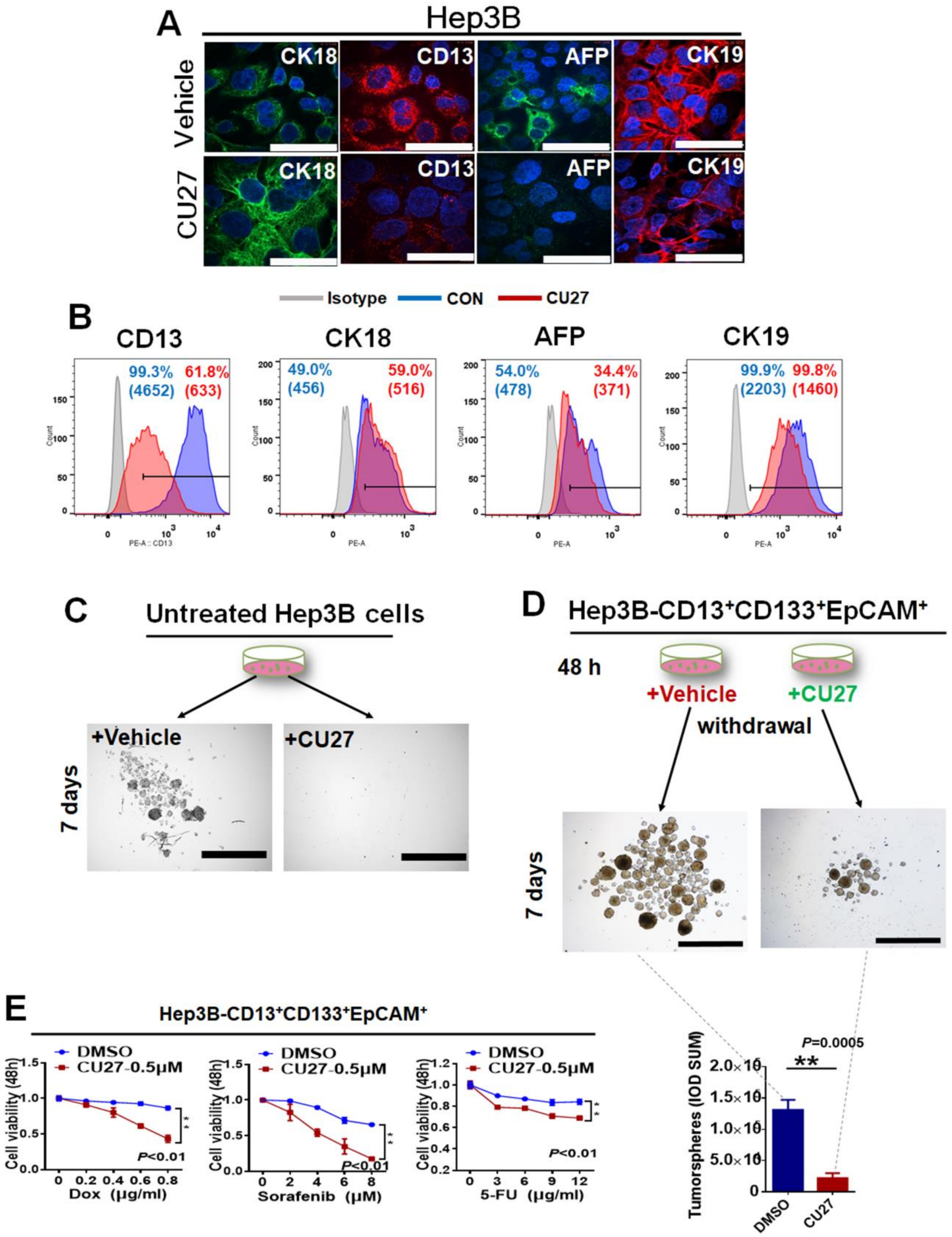

Figure S5. CU27 promotes differentiation, inhibits tumorsphere formation and increases the sensitivity of chemical drugs in Hep3B cells.

(A,B) Representative immunofluorescence images **(A)** and flow cytometry analysis **(B)** of expression levels of CK18, CK19 and AFP besides CD13 in Hep3B cells treated with CU27 (10 μM, 48h, n=3). Bars, 100 μm. **(C)** Representative images of untreated HCC cells-derived tumorspheres simultaneously treated with CU27 (7 days). Bars, 1000 μm. **(D)** Representative images and quantitative analysis of tumorspheres of CDM-cultured sorting $CD13^{+}/CD133^{+}/EpCAM^{+}$ Hep3B cells after a 48h pre-treatment with CU27 or vehicle (DMSO) before proceeding to tumorsphere formation. Bars, 1000 μm. Mean ± SD, $^{**}p < 0.01$, data analyzed by Student's t test. **(E)** Chemoresistance assay of doxorubicin (Dox), sorafenib and 5-FU in $CD13^{+}/CD133^{+}/EpCAM^{+}$ subset from Hep3B cells treated with vehicle or CU27 (0.5 μM) in CDM culture, n=3. Means ± SD; $^{*}p < 0.05$, $^{**}p < 0.01$; two-way ANOVA.

Figure. S6.

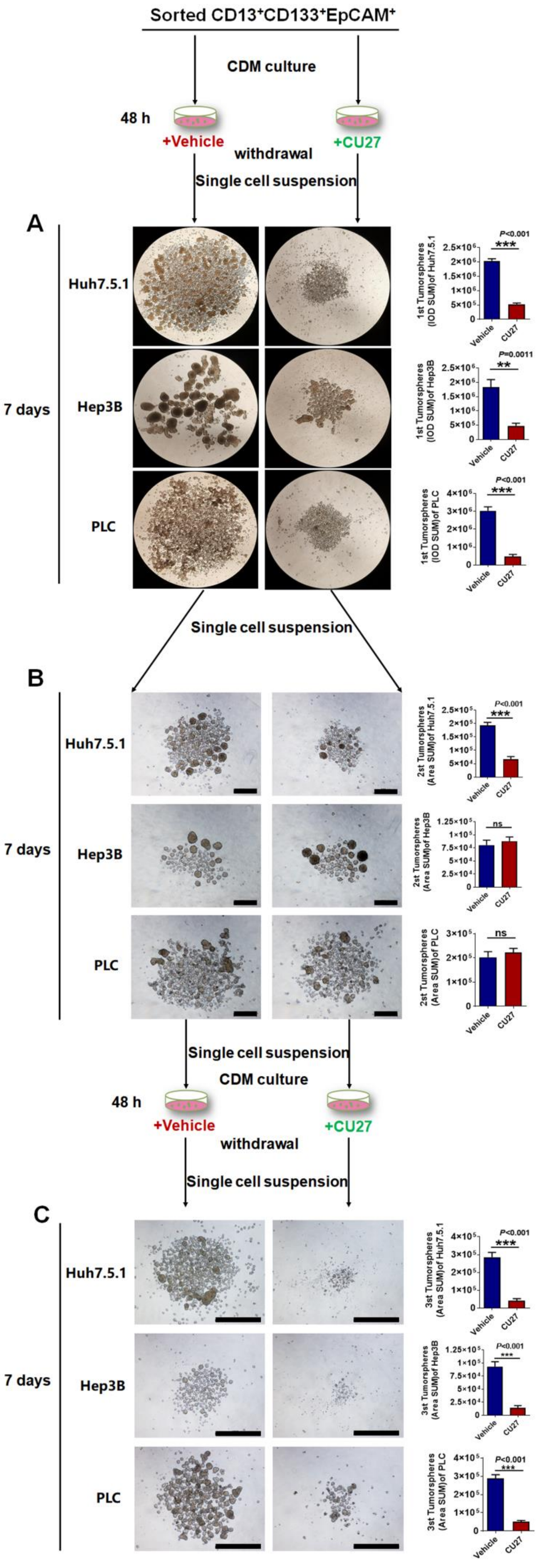

Sorted CD13+CD133+EpCAM+
CDM culture
48 h
+Vehicle
withdrawal
+CU27
Single cell suspension
A
7 days
Huh7.5.1
Hep3B
PLC
1st Tumorspheres (IOD SUM)of Huh7.5.1
P<0.001
***
1st Tumorspheres (IOD SUM)of Hep3B
P=0.0011
**
1st Tumorspheres (IOD SUM)of PLC
Vehicle
CU27
B
2st Tumorspheres (Area SUM)of Huh7.5.1
2st Tumorspheres (Area SUM)of Hep3B
2st Tumorspheres (Area SUM)of PLC
ns
C
3st Tumorspheres (Area SUM)of Huh7.5.1
3st Tumorspheres (Area SUM)of Hep3B
3st Tumorspheres (Area SUM)of PLC

Figure. S6. CU27 inhibits tumorsphere formation in a serial tumorsphere formation assay.

The primary tumorsphere formation was performed as well as that in Figure 2D, but cell seeded at a higher density (20000 cells/well) to ensure enough tumorspheres **(A, left)**, which would be digested into single cell suspensions used for secondary tumorsphere formation. **(A, right)** A quantitative statistical analysis for tumorspheres in two group. Mean ± SD, $^{**}p < 0.01$, $^{***}p < 0.001$, data analyzed by Student's t test. Then, primary tumorspheres were digested from each group by 1:1 of trypsin and accutase into single cell suspensions, and proceeded to the secondary tumorsphere formation at the same number cells (3000 cells per well) without CU27 treatment for another 7 days (**B, left**), Bars, 500 μm. **(B, right)** A quantitative statistical analysis for tumorspheres in two group. Mean ± SD, $^{***}p < 0.001$, ns indicates no significant difference, analyzed by Student's t testCU27. **(C)** The third round of tumorsphere formation, similar to the primary tumorsphere formation, using secondary tumorspheres-derived single cells instead of sorted $CD13^{+}$/$CD133^{+}$/$EpCAM^{+}$ CSC cells (C, left), Bars, 1000 μm. **(C, right)** A quantitative statistical analysis for tumorspheres in two group. Mean ± SD, $^{***}p < 0.001$, data analyzed by Student's t test.CU27.

Figure. S7.

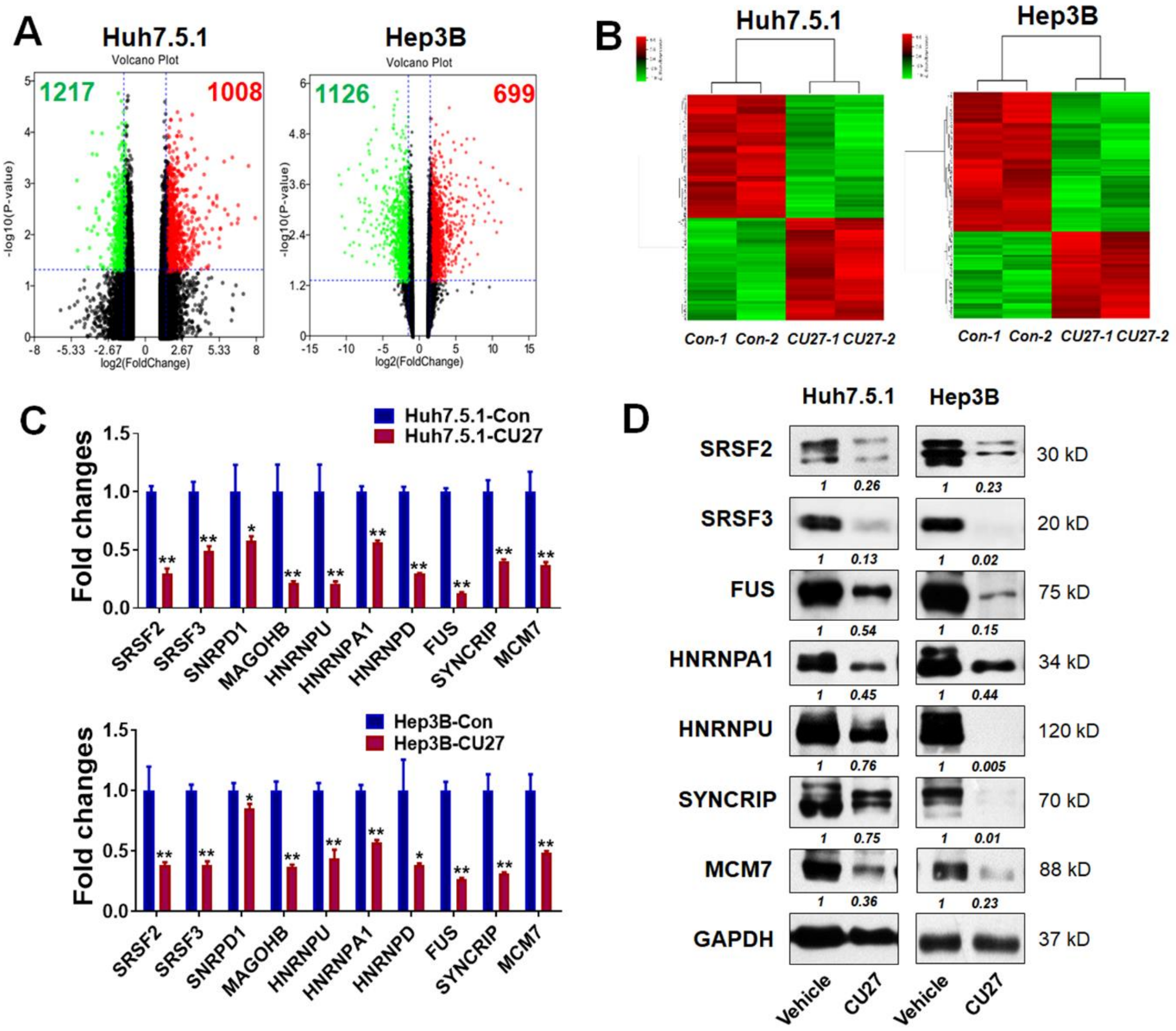

Figure S7. Analysis and verification of cDNA microarray in CU27-treated HCC cells.

(A) Volcano plots of differential genes in the cDNA microarray of CU27-treated Huh7.5.1 and Hep3B cells. Screening cutoff: fold change (FC)>=1.5, p<0.05). Red, up-regulated genes; Green, down-regulated genes. **(B)** Heat map of differentially expressed genes identified by cDNA microarray of CU27-treated HCC cells. Green, down-regulated genes; red, up-regulated genes. **(C)** The mRNA expression of selected common differential genes (SRSF2, SRSF3, SNRPD1, MAGOHB, FUS, HNRNPA1, HNRNPD, HNRNPU, SYNCRIP and MCM-7) in CU27-treated HCC cells were validated by qRT-PCR. Mean ± SD, $*p < 0.05$, $**p < 0.01$, data analyzed by Student’s t test. **(D)** The protein expression of selected common differential genes above were analyzed by western blot in CU27-treated HCC cells. Relative value=each protein band’s grayscale value/GAPDH band’s grayscale value. Normalized

relative value=each protein band's relative value in different group/each protein band's relative value in control group. Each protein's normalized relative value was indicated under the protein band. The normalized relative value for each protein in control group is 1.

Figure. S8.

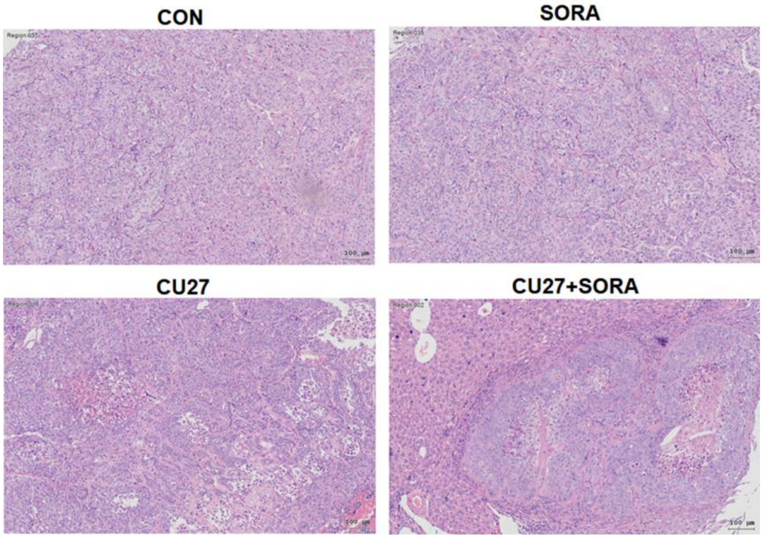

Figure S8. H&E staining of liver tumor tissues from the mice treated with vehicle (CON), CU27, Sorafenib (SORA), and CU27 and sorafenib combined therapy (CU27+SORA), respectively. Bars, 100 μm.

Figure. S9.

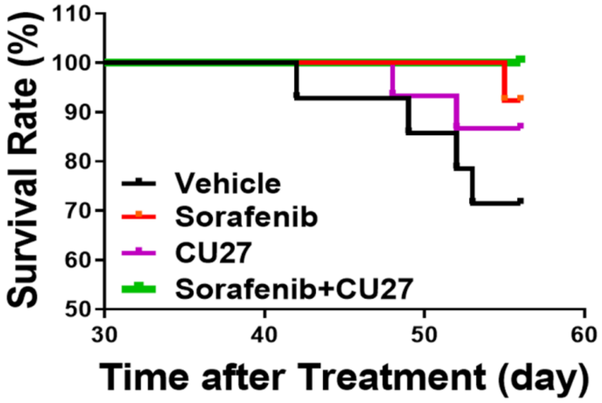

Figure S9. The survival curve of mice treated with CU27 and sorafenib combined therapy. The survival curve (in 8 weeks) of xenografted mice is shown for each group.

Figure. S10.

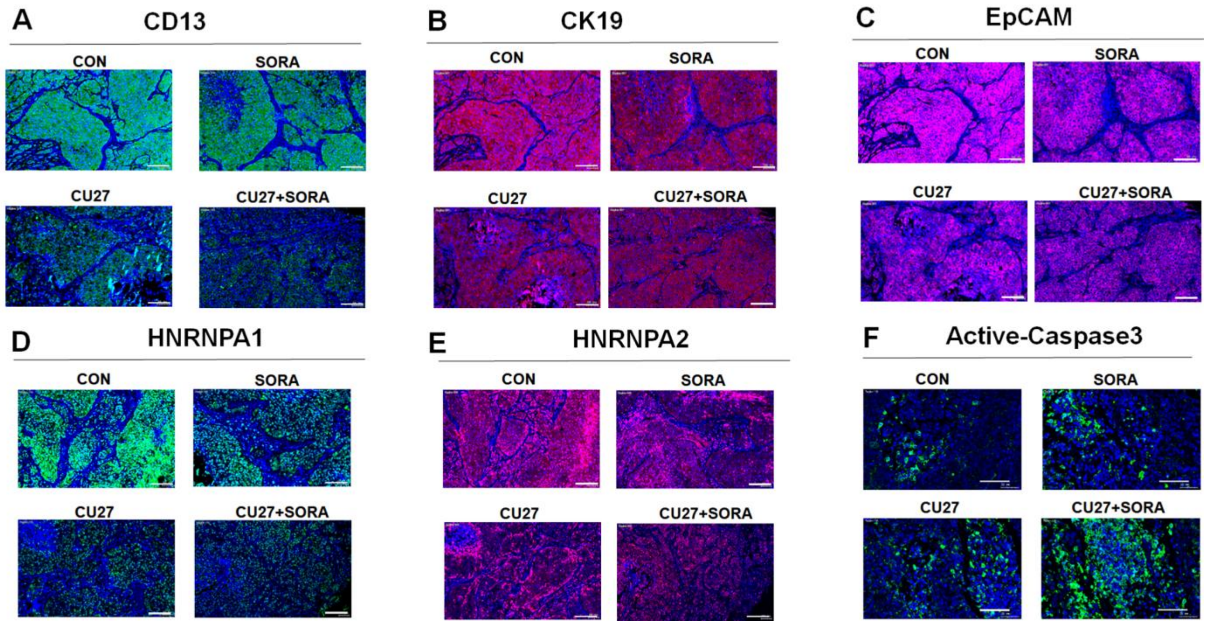

Figure S10. Representative IHC staining of CD13 (A), CK19 (B), EpCAM (C), HNRNPA1 (D), HNRNPA2 (E) and active-caspase 3 (F) in xenograft tumor tissues. These are lower magnification IHC staining photos corresponded to Figure 7C-H. Bars, 100 μm.

Figure. S11.

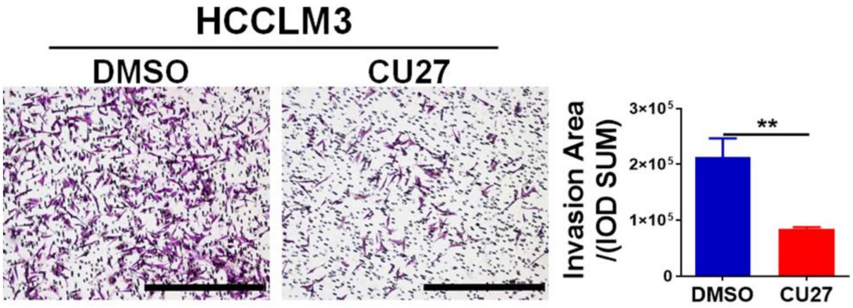

Figure S11. In vitro invasion assay of CU27-treated HCCLM3 cells. Mean ± SD, **$p < 0.01$, data analyzed by Student's t test. Bars, 1, 000 μm.

Figure. S12.

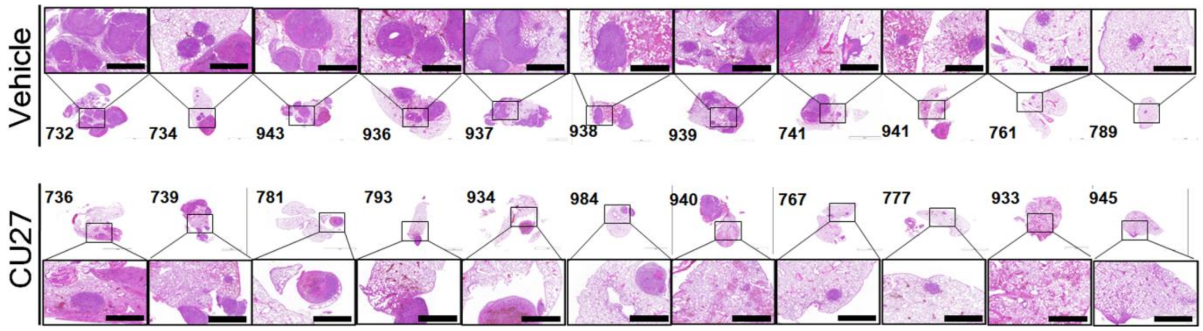

Figure S12. Representative H&E staining images of metastatic lung foci from mice in two groups. Bars, 2, 000 μm.

Figure. S13.

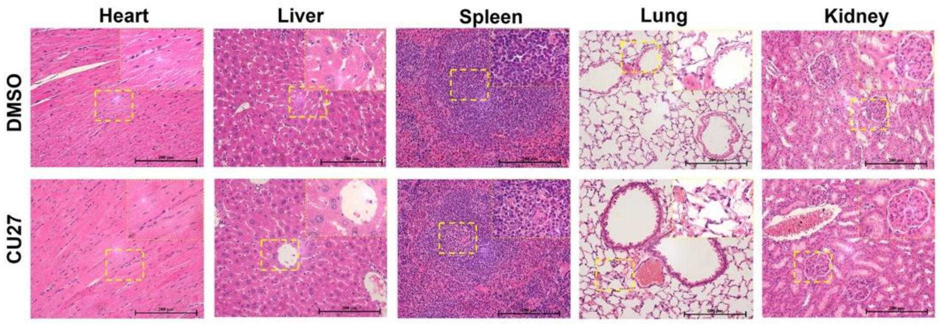

Figure S13. H&E staining of the main organs of CU27-treated mice. Representative histopathological images of vital organs (heart, liver, spleen, lung and kidney) from DMSO or CU27-treated xenografted mice. Bars, 200 μm. the enlarged pathological image showed in the upper right corner with yellow box.

Table S1. Small molecule compounds screening by cell viability assay

Number	Name	Stock cocentration	Treated concentration	Cell viability of L-02 (Mean of L-02/DMSO) (%)	Cell viability of Hep3B (Mean of Hep3B/DMSO) (%)	Relative cell viability (L-02-Hep3B) (%)
	DMSO			100	100	0
mTOR						
1	mTOR1	10mM	10μM	67.03716	44.89486	22.1423
2	mTOR2	10mM	10μM	98.95678	68.27284	30.68394
3	mTOR4	10mM	10μM	100.9368	59.52338	41.41342
4	mTOR5	10mM	10μM	100.3307	71.22107	29.10963
5	mTOR6	10mM	10μM	95.81852	68.36351	27.45501
6	mTOR7	10mM	10μM	97.84598	76.61197	21.23401
7	mTOR10	10mM	10μM	103.8162	90.78163	13.03457
8	mTOR11	10mM	10μM	98.53834	73.33677	25.20157
9	mTOR12	10mM	10μM	84.37701	59.77434	24.60267
10	mTOR13	10mM	10μM	73.37982	52.58647	20.79335
11	mTOR15	10mM	10μM	71.55941	48.59138	22.96803
12	mTOR16	10mM	10μM	96.43683	72.58299	23.85384
13	mTOR17	10mM	10μM	98.42762	83.52141	14.90621
14	mTOR18	10mM	10μM	107.2643	105.8708	1.3935
15	mTOR19	10mM	10μM	103.6235	86.95598	16.66752
16	mTOR20	10mM	10μM	101.3955	82.3042	19.0913
17	mTOR21	10mM	10μM	97.41173	65.63967	31.77206
18	mTOR22	10mM	10μM	70.03666	47.12047	22.91619
19	mTOR24	10mM	10μM	87.47285	87.26097	0.21188
20	mTOR25	10mM	10μM	77.18311	60.42645	16.75666
21	mTOR26	10mM	10μM	87.89128	62.85354	25.03774
22	mTOR27	10mM	10μM	87.8352	75.795	12.0402
23	mTOR28	10mM	10μM	106.8934	111.0777	-4.1843
24	mTOR29	10mM	10μM	98.33056	40.33925	57.99131
25	mTOR31	10mM	10μM	96.20388	96.59476	-0.39088
26	mTOR32	10mM	10μM	104.7063	109.2615	-4.5552
27	mTOR33	10mM	10μM	102.0533	95.41052	6.64278
28	mTOR34	10mM	10μM	72.22157	79.88626	-7.66469
29	mTOR35	10mM	10μM	77.35063	89.54702	*-12.19639*
30	mTOR36	10mM	10μM	60.86849	53.85222	7.01627
31	mTOR37	10mM	10μM	92.83484	54.16179	38.67305
32	mTOR39	10mM	10μM	90.69666	55.85984	34.83682
33	mTOR40	10mM	10μM	108.0099	95.56897	12.44093
34	mTOR41	10mM	10μM	103.0685	67.52364	35.54486
35	mTOR42	10mM	10μM	104.8932	79.55288	25.34032
36	mTOR43	10mM	10μM	107.8654	95.15682	12.70858
37	mTOR44	10mM	10μM	107.2917	94.98371	12.30799
38	mTOR45	10mM	10μM	106.8711	82.69895	24.17215
39	mTOR46	10mM	10μM	24.0729	11.38629	12.68661
40	mTOR47	10mM	10μM	30.38608	17.3029	13.08318
41	mTOR48	10mM	10μM	43.34603	9.603973	33.742057
42	mTOR49	10mM	10μM	88.87625	44.68971	44.18654
43	mTOR50	10mM	10μM	40.54784	22.47216	18.07568
44	mTOR52	10mM	10μM	83.16413	68.6053	14.55883
45	mTOR53	10mM	10μM	26.462	14.07166	12.39034
46	mTOR54	10mM	10μM	42.15112	39.79155	2.35957
47	mTOR55	10mM	10μM	85.78257	13.11731	72.66526
48	mTOR56	10mM	10μM	10.89079	10.97414	-0.08335
49	mTOR57	10mM	10μM	33.25328	20.47919	12.77409
50	mTOR58	10mM	10μM	59.71385	34.95751	24.75634
51	mTOR59	10mM	10μM	26.11977	11.81858	14.30119
52	mTOR60	10mM	10μM	39.82169	29.04089	10.7808
53	mTOR61	10mM	10μM	91.01228	75.92139	15.09089
54	mTOR62	10mM	10μM	39.37019	23.76539	15.6048
55	mTOR63	10mM	10μM	12.04112	10.98146	1.05966
56	mTOR64	10mM	10μM	76.05003	34.00682	42.04321
57	mTOR68	10mM	10μM	59.58228	57.75389	1.82839
58	mTOR69	10mM	10μM	62.02386	54.40175	7.62211
59	mTOR70	10mM	10μM	81.77582	63.1045	18.67132
60	mTOR71	10mM	10μM	66.44474	52.2421	14.20264
61	mTOR72	10mM	10μM	81.06117	52.29705	28.76412
62	mTOR73	10mM	10μM	85.52231	77.2476	8.27471
63	mTOR74	10mM	10μM	18.39528	9.957505	8.437775
64	mTOR75	10mM	10μM	27.92005	10.72135	17.1987
65	mTOR76	10mM	10μM	29.58372	22.39889	7.18483
66	mTOR77	10mM	10μM	33.55525	21.60024	11.95501
67	mTOR78	10mM	10μM	9.774964	8.647789	1.127175
68	mTOR79	10mM	10μM	16.90416	9.189993	7.714167
69	mTOR80	10mM	10μM	25.49859	18.33786	7.16073
70	mTOR81	10mM	10μM	25.65533	11.20311	14.45222
71	mTOR82	10mM	10μM	20.25163	11.61159	8.64004
72	mTOR83	10mM	10μM	39.71097	42.19392	-2.48295
73	mTOR84	10mM	10μM	26.83442	15.64332	11.1911
74	mTOR85	10mM	10μM	68.29821	67.71964	0.57857
75	mTOR86	10mM	10μM	30.26385	32.65681	-2.39296
76	mTOR87	10mM	10μM	30.8649	29.73605	1.12885
77	mTOR88	10mM	10μM	26.97677	23.79104	3.18573
78	mTOR89	10mM	10μM	25.80918	17.4989	8.31028
79	mTOR90	10mM	10μM	24.6833	16.20567	8.47763
80	mTOR91	10mM	10μM	86.97533	68.47342	18.50191
81	mTOR92	10mM	10μM	77.45487	62.03108	15.42379
82	mTOR93	10mM	10μM	95.94721	93.39099	2.55622
83	mTOR94	10mM	10μM	48.6275	44.15025	4.47725
84	mTOR95	10mM	10μM	100.325	101.1888	-0.8638
85	mTOR96	10mM	10μM	97.39017	86.4092	10.98097
86	mTOR97	10mM	10μM	44.19081	25.84262	18.34819
87	mTOR98	10mM	10μM	38.36508	26.15402	12.21106
88	YL	10mM	10μM	66.19526	53.83024	12.36502
89	PI	10mM	10μM	25.7833	14.55708	11.22622
90	GDC-0941	10mM	10μM	26.60867	12.58243	14.02624

**CU**						
**1**	**CU-1**	10mM	10μM	24.5574	18.32122	6.23618
**2**	**CU-2**	10mM	10μM	25.97844	43.25531	*-17.27687*
**3**	**CU-3**	10mM	10μM	31.75387	15.27036	16.48351
**4**	**CU-4**	10mM	10μM	21.0701	15.44396	5.62614
**5**	**CU-5**	10mM	10μM	48.10527	41.93723	6.16804
**6**	**CU-6**	10mM	10μM	37.4396	43.20226	-5.76266
**7**	**CU-7**	10mM	10μM	58.49633	44.20689	14.28944
**8**	**CU-8**	10mM	10μM	8.685182	14.11624	-5.431058
**9**	**CU-9**	10mM	10μM	97.42228	60.51885	**36.90343**
**10**	**CU-10**	10mM	10μM	7.556848	13.82691	-6.270062
**11**	**CU-11**	10mM	10μM	55.36155	38.42987	16.93168
**12**	**CU-12**	10mM	10μM	5.832873	3.250176	2.582697
**13**	**CU-13**	10mM	10μM	30.05586	5.220856	24.835004
**14**	**CU-14**	10mM	10μM	18.78747	10.24239	8.54508
**15**	**CU-15**	10mM	10μM	27.32079	3.94779	23.373
**16**	**CU-16**	10mM	10μM	69.82688	64.87814	4.94874
**17**	**CU-17**	10mM	10μM	15.39853	18.01099	-2.61246
**18**	**CU-18**	10mM	10μM	24.56369	19.26959	5.2941
**19**	**CU-19**	10mM	10μM	23.59587	5.114767	18.481103
**20**	**CU-20**	10mM	10μM	18.78196	18.27943	0.50253
**21**	**CU-22**	10mM	10μM	27.43724	24.24612	3.19112
**22**	**CU-23**	10mM	10μM	52.74686	18.90953	33.83733
**23**	**CU-24**	10mM	10μM	54.32842	22.06969	32.25873
**24**	**CU-25**	10mM	10μM	60.7113	20.00257	**40.70873**
**25**	**CU-26**	10mM	10μM	38.54591	19.07348	19.47243
**26**	**CU-27**	10mM	10μM	79.37838	30.94579	**48.43259**
**27**	**CU-28**	10mM	10μM	54.89731	20.66482	34.23249
**28**	**CU-29**	10mM	10μM	108.9015	50.58187	**58.31963**
**29**	**CU-30**	10mM	10μM	26.63624	32.81359	-6.17735
**30**	**CU-31**	10mM	10μM	25.02163	17.37285	7.64878
**31**	**CU-32**	10mM	10μM	35.33401	48.09039	*-12.75638*
**32**	**CU-33**	10mM	10μM	23.86969	52.44003	*-28.57034*
**33**	**CU-34**	10mM	10μM	56.93759	96.62602	*-39.68843*
**34**	**CU-36**	10mM	10μM	66.79281	88.97316	*-22.18035*
**35**	**CU-37**	10mM	10μM	54.73994	67.38891	*-12.64897*
**36**	**CU-38**	10mM	10μM	59.24619	55.49249	3.7537
**37**	**CU-39**	10mM	10μM	44.11755	60.93195	*-16.8144*
**38**	**CU-40**	10mM	10μM	26.37343	53.38518	*-27.01175*
**39**	**CU-41**	10mM	10μM	42.94279	53.51056	*-10.56777*
**40**	**CU-42**	10mM	10μM	103.7343	71.57781	32.15649
**41**	**CU-43**	10mM	10μM	29.49091	82.04685	*-52.55594*
**AKT**						
**1**	**H89 2HCL**	10mM	10μM	91.53512	56.95362	34.5815
**2**	**A-674563**	10mM	10μM	10.70265	21.69677	*-10.99412*
**3**	**CCT128930**	10mM	10μM	97.58909	34.43386	**63.15523**
**4**	**ATI13148**	10mM	10μM	105.7471	55.36872	**50.37838**
**5**	**AZD5363**	10mM	10μM	50.62868	36.10878	14.5199
**6**	**GSK458**	10mM	10μM	9.810369	18.00295	-8.192581
**7**	**AZD2014**	10mM	10μM	32.35974	29.88811	2.47163
**8**	**GSK690693**	10mM	10μM	49.34927	63.77385	*-14.42458*
**9**	**FRAX597**	10mM	10μM	40.06766	19.36925	20.69841
**10**	**GDC-0349**	10mM	10μM	26.81721	26.30681	0.5104
**11**	**Dactolisib**	10mM	10μM	27.21378	21.03452	6.17926
**12**	**GSK105961**	10mM	10μM	13.54945	17.92258	-4.37313
**13**	**PI-103**	10mM	10μM	22.04579	22.82196	-0.77617
**14**	**XL388**	10mM	10μM	33.22842	34.64925	-1.42083
**15**	**WYE-687**	10mM	10μM	31.40766	21.64533	9.76233
**16**	**OSI-027**	10mM	10μM	28.29018	26.4129	1.87728
**17**	**KU-0063797**	10mM	10μM	29.952	26.72474	3.22726
**18**	**PP121**	10mM	10μM	23.3189	22.12113	1.19777
**19**	**Palomid529**	10mM	10μM	58.05963	62.35772	-4.29809
**20**	**AZD8055**	10mM	10μM	22.97112	22.14685	0.82427
**21**	**Gedatolisib**	10mM	10μM	25.82421	22.48762	3.33659
**22**	**WYE-354**	10mM	10μM	39.96459	33.97414	5.99045
**23**	**Torkinib**	10mM	10μM	46.5788	44.8386	1.7402
**24**	**Apitolisib**	10mM	10μM	37.08946	29.35124	7.73822
**25**	**4EG-1**	10mM	10μM	98.46092	117.4323	*-18.97138*
**26**	**BGT226**	10mM	10μM	10.69636	17.51752	-6.82116
**27**	**XL888**	10mM	10μM	50.43354	39.15642	11.27712
**28**	**WYE-12513**	10mM	10μM	45.12235	27.16838	17.95397
**29**	**WAY-600**	10mM	10μM	36.86993	23.19809	13.67184
**30**	**Torin2**	10mM	10μM	13.00023	17.29248	-4.29225
**31**	**Voxtalisib**	10mM	10μM	26.91635	23.12093	3.79542
**32**	**SNX-2112**	10mM	10μM	49.30521	37.11501	12.1902
**33**	**Torin1**	10mM	10μM	26.63781	24.0468	2.59101
**34**	**PHA-767491**	10mM	10μM	22.2189	26.7376	-4.5187
**35**	**JNJ-646191**	10mM	10μM	85.77149	44.74859	**41.0229**
**36**	**SC-514**	10mM	10μM	102.2338	75.8519	26.3819
**37**	**PX-12**	10mM	10μM	69.52395	64.79937	4.72458
**38**	**Orlisat**	10mM	10μM	102.0599	66.8006	**35.2593**
**39**	**JNJ2685416**	10mM	10μM	37.99826	17.65575	20.34251
**40**	**NCT-501**	10mM	10μM	95.94144	64.68364	31.2578
**41**	**NU7441**	10mM	10μM	83.16939	37.03143	**46.13796**
**LX**						
**1**	**LX-2-23**	10mM	10μM	23.26855	23.80247	-0.53392
**2**	**LX-2-45**	10mM	10μM	35.78566	34.21525	1.57041
**3**	**LX-2-41**	10mM	10μM	54.32527	56.48426	-2.15899
**4**	**LX-2-46**	10mM	10μM	42.04579	45.89306	-3.84727
**5**	**LX-2-49**	10mM	10μM	28.83861	26.67009	2.16852
**6**	**LX-2-50**	10mM	10μM	28.50342	26.59615	1.90727
**7**	**LX-3-3**	10mM	10μM	28.62223	23.45849	5.16374
**8**	**LX-3-7**	10mM	10μM	30.52167	24.82157	5.7001
**9**	**LX-3-10**	10mM	10μM	27.50649	32.46639	-4.9599
**10**	**LX-3-16**	10mM	10μM	52.40537	46.56978	5.83559
**11**	**LX-3-22**	10mM	10μM	91.95765	77.4352	14.52245
**12**	**LX-3-292**	10mM	10μM	42.86568	22.32688	20.5388

LXY						
1	LXY-3-1	10mM	10μM	86.22177955	91.11205114	-4.890271589
2	LXY-3-2	10mM	10μM	94.98094578	95.86388419	-0.882938407
3	LXY-4-34	10mM	10μM	95.47606675	93.13193269	2.344134053
4	LXY-4-42	10mM	10μM	92.86477279	89.98636962	2.878403175
5	LXY-5-5	10mM	10μM	90.27080085	90.68668923	-0.415888373
6	LXY-5-6	10mM	10μM	88.55303424	91.00629818	-2.453263936
7	LXY-5-8	10mM	10μM	92.30036376	91.01804851	1.282315257
8	LXY-5-9	10mM	10μM	92.61793406	92.94980259	-0.331868534
9	LXY-5-10	10mM	10μM	89.38737802	90.27307765	-0.885699622
10	LXY-5-11	10mM	10μM	90.94058548	89.45760481	1.482980671
11	LXY-5-13	10mM	10μM	89.34046423	98.20689979	-8.866435563
12	LXY-5-15	10mM	10μM	73.14365726	85.92780598	*-12.78414872*
13	LXY-5-16	10mM	10μM	89.08279924	93.29878737	-4.215988128
14	LXY-5-17	10mM	10μM	90.6345632	103.7366046	*-13.10204143*
15	LXY-5-18	10mM	10μM	71.30752353	82.95497274	*-11.64744921*
16	LXY-5-19	10mM	10μM	90.43969051	100.0070502	-9.567359684
17	LXY-5-20	10mM	10μM	91.36064438	97.50188005	-6.141235674
18	LXY-5-22	10mM	10μM	91.2769213	98.75446513	-7.477543825
19	LXY-5-26	10mM	10μM	90.22460881	92.88165069	-2.657041875
20	LXY-5-29	10mM	10μM	72.7712339	69.65595037	3.115283538
21	LXY-5-30	10mM	10μM	95.19169698	82.45440872	12.73728826
22	LXY-5-31	10mM	10μM	64.58225071	58.6999436	5.882307109
23	LXY-5-34	10mM	10μM	93.04809747	79.76005828	13.28803918
24	LXY-5-35	10mM	10μM	88.60500029	97.19872156	-8.593721276
25	LXY-5-36	10mM	10μM	90.40793348	88.60453093	1.803402557
26	LXY-5-50	10mM	10μM	89.43934407	89.42940402	0.00994005
27	LXY-6-9	10mM	10μM	82.94936197	83.3356834	-0.386321427
28	LXY-6-14	10mM	10μM	91.07194411	90.01927054	1.052673568
29	LXY-6-15	10mM	10μM	91.72007622	86.66925174	5.050824478
30	LXY-6-16	10mM	10μM	85.2156591	87.75145704	-2.535797938
31	LXY-6-18	10mM	10μM	90.66487672	94.60894905	-3.944072326
32	LXY-6-19	10mM	10μM	91.55840406	103.5838503	*-12.02544628*
33	LXY-6-20	10mM	10μM	93.36133726	96.86501222	-3.503674961
34	LXY-6-26	10mM	10μM	89.40903054	93.33286332	-3.923832776
35	LXY-6-29	10mM	10μM	90.43391651	93.77702576	-3.343109249
36	LXY-6-30	10mM	10μM	84.91829782	84.80682459	0.111473232
37	LXY-6-31	10mM	10μM	90.6432242	97.464279	-6.821054795
38	LXY-6-35	10mM	10μM	88.9413361	97.98364354	-9.042307437
39	LXY-3-38	10mM	10μM	90.67642474	86.19101335	4.485411387
40	LXY-5-47	10mM	10μM	84.73641665	91.60321489	-6.866798238
41	LXY-6-46	10mM	10μM	85.635718	91.93457417	-6.298856171
42	LXY-6-47	10mM	10μM	87.08354986	93.50911826	-6.425568397
43	LXY-6-49	10mM	10μM	87.9741902	95.68762925	-7.713439058
44	LXY-6-50	10mM	10μM	84.87932329	93.30113743	-8.421814145
45	LXY-7-3	10mM	10μM	93.17223858	102.7660274	-9.593788867
46	LXY-7-4	10mM	10μM	96.26999249	99.34198158	-3.071989082
47	LXY-7-5	10mM	10μM	97.34973151	98.43250611	-1.082774601
48	LXY-7-6	10mM	10μM	93.41907731	94.45619477	-1.03711746
49	LXY-7-9	10mM	10μM	88.05935678	90.41173153	-2.352374753
50	LXY-7-10	10mM	10μM	87.98573821	87.6927054	0.293032811
51	LXY-7-14	10mM	10μM	97.04803973	84.59531867	12.45272106
52	LXY-7-15	10mM	10μM	67.25128472	56.22297424	11.02831047
53	LXY-7-17	10mM	10μM	89.05537271	87.40834743	1.647025278
54	LXY-7-23	10mM	10μM	33.18176569	43.62427148	*-10.44250579*
55	LXY-7-25	10mM	10μM	85.09296149	89.01344238	-3.920480889
56	LXY-7-26	10mM	10μM	87.8161268	92.67249483	-4.856368033
57	LXY-7-27	10mM	10μM	50.15445465	48.5570596	1.597395048
58	LXY-7-30	10mM	10μM	79.81840753	91.08150028	*-11.26309275*
59	LXY-7-36	10mM	10μM	88.25134246	92.54794134	-4.296598886
60	LXY-7-37	10mM	10μM	76.78994168	88.76903553	*-11.97909385*
61	LXY-7-45	10mM	10μM	82.19441076	83.18292912	-0.988518359
62	LXY-7-46	10mM	10μM	81.00785265	78.11618725	2.891665394
63	LXY-8-3-1	10mM	10μM	74.0386281	74.97414928	-0.93552118
64	LXY-8-3-2	10mM	10μM	86.62018592	89.78426396	-3.164078036
65	LXY-8-4	10mM	10μM	87.86304059	87.24619289	0.616847698
66	LXY-8-9	10mM	10μM	89.21127086	90.38353074	-1.17225988
67	LXY-8-10	10mM	10μM	94.96795427	92.02387667	2.944077601
68	LXY-8-11	10mM	10μM	94.32126566	94.2822899	0.038975758
69	LXY-8-12	10mM	10μM	88.46353716	89.55160745	-1.088070289
70	LXY-8-13	10mM	10μM	90.35596743	97.53008084	-7.174113408
71	LXY-8-14	10mM	10μM	90.79046134	92.29413424	-1.503672893
72	LXY-8-15	10mM	10μM	92.51255846	96.35034781	-3.837789348
73	LXY-8-16	10mM	10μM	91.46168947	100.0117503	-8.550060855
74	LXY-8-17	10mM	10μM	91.21485074	90.94754653	0.267304211
75	LXY-8-18	10mM	10μM	90.20151279	99.47358526	-9.272072471
76	LXY-8-19	10mM	10μM	91.79080778	93.41276556	-1.621957774
77	LXY-8-20	10mM	10μM	92.41006987	102.3195149	-9.909445081
78	LXY-8-21	10mM	10μM	87.79230902	91.86877233	-4.076463301
79	LXY-8-23	10mM	10μM	86.703909	84.38851288	2.315396123
80	LXY-8-24	10mM	10μM	92.53421098	85.12408347	7.410127508
81	LXY-8-27	10mM	10μM	90.27513136	82.05489754	8.220233821
82	LXY-8-29	10mM	10μM	97.63843178	86.15811243	11.48031935
83	LXY-8-34	10mM	10μM	91.58727409	83.82802218	7.759251907
84	LXY-8-37	10mM	10μM	93.04809747	94.1213104	-1.073212931

HTL						
1	HTL-02-35	10mM	10µM	56.88274547	44.92795286	11.95479261
2	HTL-03-11	10mM	10µM	10.02700985	8.583485902	1.443523949
3	HTL-02-38	10mM	10µM	64.15475056	86.03565718	-21.88090662
4	HTL-02-41	10mM	10µM	23.46202733	8.56569366	14.89633367
5	HTL-02-42	10mM	10µM	36.27740705	23.57726243	12.70014462
6	HTL-02-34	10mM	10µM	40.7435653	42.91870038	-2.175135083
7	HTL-03-07	10mM	10µM	23.46838259	25.0946865	-1.626303913
8	HTL-03-04	10mM	10µM	15.85001589	10.41100333	5.439012556
9	HTL-03-12	10mM	10µM	7.546870035	7.942965189	-0.396095154
10	HTL-03-15	10mM	10µM	8.004448681	9.348552309	-1.344103628
11	HTL-03-16	10mM	10µM	6.050206546	8.291184783	-2.240978237
12	HTL-03-18	10mM	10µM	5.27804258	8.601278144	-3.323235564
13	HTL-03-22-1	10mM	10µM	11.43628853	33.449415	-22.01312648
14	HTL-03-24	10mM	10µM	7.615189069	8.298810029	-0.68362096
15	HTL-03-25	10mM	10µM	5.927867811	8.044635143	-2.116767333
16	HTL-03-26	10mM	10µM	27.43883063	41.17633154	-13.73750091
17	HTL-03-22-2	10mM	10µM	6.984429616	8.054802139	-1.070372523
18	HTL-03-33	10mM	10µM	7.14013346	7.978549673	-0.838416213
19	HTL-03-34	10mM	10µM	5.382904353	7.62778833	-2.244883977
20	HTL-03-36	10mM	10µM	6.709564665	7.881963216	-1.172398552
21	HTL-03-37	10mM	10µM	5.945344773	8.064969134	-2.119624361
22	HTL-03-39	10mM	10µM	5.111217032	7.704040796	-2.592823764
23	HTL-03-40	10mM	10µM	5.317762949	7.889588463	-2.571825514
24	HTL-03-41	10mM	10µM	6.549094376	8.067510883	-1.518416508
25	HTL-03-42	10mM	10µM	5.83730537	8.504691687	-2.667386317
26	HTL-03-43	10mM	10µM	5.346361614	7.894671961	-2.548310346
27	HTL-03-45	10mM	10µM	17.15919924	13.08492313	4.074276104
28	HTL-03-46	10mM	10µM	13.69240547	11.24469696	2.447708507
29	HTL-04-42	10mM	10µM	69.12932952	55.48129413	13.64803539
30	HTL-5-21	10mM	10µM	22.56911344	34.65674571	-12.08763227
31	HTL-5-23	10mM	10µM	84.83317445	96.15435939	-11.32118494
32	HTL-5-25	10mM	10µM	91.55068319	88.85572754	2.694955652
33	HTL-5-26	10mM	10µM	86.85811884	108.9749406	-22.1168218
34	HTL-5-27	10mM	10µM	92.45154115	78.15369396	14.29784719
35	HTL-5-28	10mM	10µM	93.16412456	108.7423706	-15.57824606
36	HTL-5-29	10mM	10µM	92.12424531	109.4324554	-17.30821013
37	HTL-5-30	10mM	10µM	68.62170321	63.56278463	5.058918581
38	HTL-5-32	10mM	10µM	65.43771846	52.31554592	13.12217254
39	HTL-5-33	10mM	10µM	93.85923101	96.94230154	-3.083070525
40	HTL-5-34	10mM	10µM	85.97870988	85.85519301	0.123516873
41	HTL-5-35	10mM	10µM	60.84524944	52.11728951	8.727959934
42	HTL-5-36	10mM	10µM	94.89672704	50.87818694	44.0185401
43	HTL-5-44	10mM	10µM	29.66158246	26.58160958	3.079972877
44	HTL-6-1	10mM	10µM	64.84191293	66.35362488	-1.511711944
45	HTL-6-12	10mM	10µM	27.8662218	48.33516721	-20.46894541
46	HTL-6-16	10mM	10µM	78.09977757	83.46849083	-5.368713263
47	HTL-6-17	10mM	10µM	66.79377185	63.78010416	3.01366769
48	HTL-6-18	10mM	10µM	81.94470925	96.39074204	-14.44603279
49	HTL-6-19	10mM	10µM	51.49030823	70.01755586	-18.52724763
50	HTL-6-20	10mM	10µM	81.9717191	98.92486565	-16.95314655
51	HTL-6-21	10mM	10µM	69.44073721	83.68962298	-14.24888577
52	HTL-6-22	10mM	10µM	43.65109628	41.27800149	2.373094788
53	HTL-6-23	10mM	10µM	69.61550683	64.86034742	4.75515941
54	HTL-6-24	10mM	10µM	75.30664125	81.49863546	-6.191994216
55	HTL-6-25	10mM	10µM	64.97775659	57.53502721	7.442729388
56	HTL-6-26	10mM	10µM	76.62535748	66.76920082	9.856156668
57	HTL-6-27	10mM	10µM	79.40896092	91.83846983	-12.42950891
58	HTL-6-28	10mM	10µM	72.38957738	78.09523374	-5.705656363
59	HTL-6-29	10mM	10µM	33.95614871	41.43050643	-7.474357713
60	HTL-6-30	10mM	10µM	63.36352081	37.85426578	25.50925503
61	HTL-6-31	10mM	10µM	87.13536702	98.31484592	-11.17947891
62	HTL-6-32	10mM	10µM	62.93295202	92.09264471	-29.15969269
63	HTL-6-33	10mM	10µM	73.41277407	98.30467893	-24.89190486
64	HTL-6-34	10mM	10µM	93.7718462	107.1677572	-13.395911
65	HTL-6-35	10mM	10µM	83.87511916	77.19291289	6.682206269
66	HTL-6-38	10mM	10µM	86.58722593	111.6996954	-25.11246949
67	HTL-6-45-1	10mM	10µM	15.10486177	8.40810523	6.696756543
68	HTL-6-45-2	10mM	10µM	29.03241182	30.74245247	-1.710040647
69	HTL-6-47	10mM	10µM	23.76231331	17.9574557	5.804857615
70	HTL-6-48	10mM	10µM	24.40260566	32.07432887	-7.671723214
71	HTL-6-49	10mM	10µM	18.83539879	16.80095997	2.034438825
72	HTL-6-50	10mM	10µM	23.31744519	40.79506921	-17.47762402
73	HTL-7-1	10mM	10µM	27.10041309	38.27365434	-11.17324125
74	HTL-7-2	10mM	10µM	74.05306641	52.63707715	21.41598926
75	HTL-7-3	10mM	10µM	15.51795361	15.10561348	0.412340129
76	HTL-7-4	10mM	10µM	27.77565936	24.86847085	2.907188507
77	HTL-7-5	10mM	10µM	23.82427709	19.91714407	3.907133019
78	HTL-7-6	10mM	10µM	18.99269145	16.09689553	2.895795919
79	HTL-7-7	10mM	10µM	23.38735303	14.74976864	8.637584397
80	HTL-7-8	10mM	10µM	22.54369241	22.66985809	-0.126165681
81	HTL-7-11	10mM	10µM	19.20400381	11.53699808	7.667005735
82	HTL-7-12	10mM	10µM	79.41531617	91.79271835	-12.37740217
83	HTL-7-13	10mM	10µM	37.23705116	32.96394097	4.273110188
84	HTL-7-14	10mM	10µM	27.19733079	42.14219611	-14.94486532
85	HTL-7-15	10mM	10µM	28.71306006	26.71632227	1.996737785
86	HTL-7-16	10mM	10µM	20.49888783	21.72941101	-1.230523178
87	HTL-7-17	10mM	10µM	17.09246902	17.49739916	-0.404930137
88	HTL-7-18	10mM	10µM	30.62758182	18.1811296	12.44645223
89	HTL-7-19	10mM	10µM	24.27073403	12.81549775	11.45523628
90	HTL-7-20	10mM	10µM	11.43787734	15.77155168	-4.333674336
91	HTL-7-21	10mM	10µM	23.72259295	16.81875221	6.903840736
92	HTL-7-23	10mM	10µM	95.23211502	79.37582252	15.8562925
93	HTL-7-24	10mM	10µM	23.77013684	14.35420192	9.415934926
94	HTL-7-25	10mM	10µM	36.4902708	37.21329197	-0.723021171
95	HTL-7-26	10mM	10µM	35.72521508	31.63893589	4.086279191
96	HTL-7-28	10mM	10µM	29.30452105	25	4.304521046
97	HTL-7-29	10mM	10µM	25.90218835	22.68048505	3.221703294

Table S2. Ingenuity pathway analysis (IPA) of transcription factor regulators*.

TF Regulators	Predicted Activation State	Huh7.5.1-Activation z-score	Hep3B-Activation z-score
NUPR1	Activated	4.341	6.173
TP53	Activated	3.603	3.765
Myc	Inhibited	-1.97	-2.795
CDKN2A	Activated	1.97	2.433
FOXM1	Inhibited	-1.977	-2.376
FOXO3	Activated	2.215	1.832
Notch	Activated	1.067	2.213
EZH2	Inhibited	-1.245	-1.929
Hdac	Inhibited	-1.326	-1.18
CBX5	Inhibited	-0.538	-1.929
HMGA1		-2.236	N/A
BRCA1		1.444	-0.647
SMAD4	Activated	1.571	0.499
HIF1A	Activated	1.055	1.004
CCND1	Activated	0.896	1.129
TFAP2C		1.982	N/A
TWIST1		1.951	N/A
SP1	Activated	1.226	0.714
SPI1		-0.742	1.188
TP73	Activated	0.816	1.046
GLI1	Inhibited	-0.711	-1.129
SREBF1		0.323	-1.481
SMARCA4		-1.41	0.311
PPRC1		-1.671	0
TCF7L2	Inhibited	-0.555	-1.067
RELA		-1.141	0.427
SATB1		N/A	-1.51
TP63	Inhibited	-1.128	-0.282
MED1		N/A	-1.406
NCOA1		-0.152	1.172
NCOA3		N/A	-1.287
FOXO4		N/A	1.218
HNF4A		1.153	N/A
NFkB		-1.137	N/A
HLX		-1.131	N/A
KDM5B		-0.033	1.077
YAP1		0	1.095

***** These transcription factors are arranged according to the absolute value of activation Z-score, of which the higher absolute value, the more consistent with changes of downstream gene expressions by regulation of putative transcription factor.

Table S3. The docking results between the optimization compounds of In-House library and c-Myc.

The result of binding energies between the optimization compounds of In-House Library and c-Myc in non-solvent model

Ligand Name	Binding Energy (kcal/mol)	Ligand Energy (kcal/mol)	Protein Energy (kcal/mol)	Complex Energy (kcal/mol)	Entropic Energy (kcal/mol)
CU27	-70.3928	23.8249	-2771.2686	-2817.8365	19.6972
CU38	-62.4636	51.7107	-2771.2686	-2782.0215	19.0184
CU6	-67.2032	45.9927	-2771.2686	-2792.4792	19.5644
CU39	-66.3806	54.3343	-2771.2686	-2783.3149	19.2781
CU8	-77.2931	66.7260	-2771.2686	-2781.8358	20.0121
D3-361	-33.5642	89.9725	-2771.2686	-2714.8603	19.7494
D3-251	-9.4804	84.7709	-2771.2686	-2695.9781	20.2007
CU20	-59.7348	36.0894	-2771.2686	-2794.9140	19.7297
CU43	-58.6101	73.4793	-2771.2686	-2756.3995	19.3694
CU23	-55.7779	36.5825	-2771.2686	-2790.4640	19.9220
10074-G5	-20.7289	100.4902	-2771.2686	-2691.5073	19.3633

Implicit Solvent Model 1: Poisson Boltzmann with non-polar Surface Area (PBSA)

Ligand Name	Binding Energy (kcal/mol)	Ligand Energy (kcal/mol)	Protein Energy (kcal/mol)	Complex Energy (kcal/mol)	Entropic Energy (kcal/mol)
CU27	1.3949	14.3883	-5582.3130	-5566.5298	19.6972
CU38	12.9716	35.9812	-5582.3130	-5533.3601	19.0184
CU6	9.7996	34.7686	-5582.3130	-5537.7448	19.5644
CU39	7.7999	42.0932	-5582.3130	-5532.4199	19.2781
CU8	7.9069	48.8117	-5582.3130	-5525.5944	20.0121
D3-361	19.0631	73.8787	-5582.3130	-5489.3712	19.7494
D3-251	28.2516	69.6843	-5582.3130	-5484.3771	20.2007
CU20	5.3948	28.8729	-5582.3130	-5548.0453	19.7297
CU43	-16.8978	57.7381	-5582.3130	-5541.4726	19.3694
CU23	4.5296	25.2418	-5582.3130	-5552.5416	19.9220
10074-G5	-14.9025	96.2617	-5582.3130	-5500.9538	19.3633

Implicit Solvent Model 2: Generalized Born (GB)

Ligand Name	Binding Energy (kcal/mol)	Ligand Energy (kcal/mol)	Protein Energy (kcal/mol)	Complex Energy (kcal/mol)	Entropic Energy (kcal/mol)
CU27	1.1443	7.2888	-5740.3297	-5731.8966	19.6972
CU38	3.2850	28.5637	-5740.3297	-5708.4810	19.0184
CU6	1.1817	27.1544	-5740.3297	-5711.9937	19.5644

CU39	3.0179	33.6301	-5740.3297	-5703.6817	19.2781
CU8	2.7776	41.3571	-5740.3297	-5696.1950	20.0121
D3-361	6.0674	66.4818	-5740.3297	-5667.7805	19.7494
D3-251	10.4133	62.0540	-5740.3297	-5667.8625	20.2007
CU20	2.8618	22.1513	-5740.3297	-5715.3166	19.7297
CU43	-1.5146	50.6581	-5740.3297	-5691.1863	19.3694
CU23	3.9733	18.1314	-5740.3297	-5718.2251	19.9220
10074-G5	-4.4157	93.6440	-5740.3297	-5651.1015	19.3633

Table S4. The kits and reagents used in the study.

Kits & Reagents	Applications	Catalog No.	Source
FBS	cell culture	FSP500	Excell Biology
DMEM-F12	cell culture	11330-057	Life Technologies
DMEM	cell culture	D1152-1L	Sigma
PRIM-1640	cell culture	R6504-1L	Sigma
MEM basic	cell culture	C11095500BT	GIBCO
Neurobasal Medium	cell culture	21103-049	GIBCO
Pen Strep	cell culture	15140-122	GIBCO
Bovine serum albumin	cell culture	V900933-100G	Sigma
N-2	tumorshpere	17502-048	GIBCO
B-27	tumorshpere	17504-044	GIBCO
L-Glutamine	cell culture	11811-031	GIBCO
rhHGF	cell culture	294-HGN	R&D
rhBMP-4	cell culture	314-BP	R&D
EGF	tumorshpere	236-EG-200	R&D
b-FGF	tumorshpere	100-18C	Peprotech
TGF-α	cell culture	100-16A-100UG	Peprotech
Matrigel Matrix	cell culture	354234	Corning
Sorafenib	in vivo chemoresistance assay	HY-10201	MedChemExpress
CHIP Kits	ChIP	17-295	Millipore
Pierce™ IP lysis buffer	Co-IP	87787	Thermo
Protein A/G Mix Magnetic beads	Co-IP	LSKMAGAG02	Millipore
Peroxidase substrate kit	IHC	SK-4100	Vector laboratories
Heparin solution	tumorshpere	7980	Stemcell
Cell Counting Kit-8	cell counting	CK04	DOJINDO
Annexin V, FITC Apoptosis Detection Kit	apoptosis	AD10	DOJINDO
Adriamycin (Dox)	drug resistance	A603456-0025	Sangon Biotech
Sorafenib	drug resistance	sc-220125	Santa Cruz

			Biotechnology
Dual-Glo Luciferase Assay System	report vector experiment	E2920	Promega
lipofectamine 2000 reagent	cell transfection	11668-019	Invitrogen
10074-G5	c-Myc inhibitor	S8426	Selleck
10058-F4	c-Myc inhibitor	S7153	Selleck
Axyprep DNA Gel Extraction Kit	plasmid construction	AP-GX-250G	Axygenbio
Axyprep plasmid miniprep Kit	plasmid construction	AP-MN-250G	Axygenbio

Table S5. The antibodies used in the study.

Antibody	Applications	Cat. No./Source	Origin	Dilution	Molecular weight/Localization
PE-CD13	FC	555394, BD Pharmingen™	mouse	10µL/test	Cell membrane
PE-IgG Isotype	FC	555574, BD Pharmingen™	mouse	10µL/test	
PerCP-CyTM5.5-CD13	FC	561361, BD Pharmingen™	mouse	5µL/test	Cell membrane
PerCP-CyTM5.5-IgG Isotype	FC	550795, BD Pharmingen™	mouse	5µL/test	
PE-CD133	FC	130-080-801, Miltenyi Biotec	mouse	5µL/test	Cell membrane
APC-EpCAM	FC	324208, Biolegend	mouse	5µL/test	Cell membrane
APC-IgG Isotype	FC	400327, Biolegend	mouse	5µL/test	
CD13	IF	ab7417, Abcam	mouse	1:200	Cell membrane/ cytoplasm
AFP	IF, FC	NB100-1611, Novus Biologicals	rabbit	1:200 (IF) 1:400 (FC)	Cytoplasm
MCM-7	WB	sc-9966, Santa Cruz	mouse	1:1000, 1:200	88 KD/Nuclei, cytoplasm
CK18	IF, FC	ab133263, Abcam	rabbit	1:200 (IF) 1:400 (FC)	Cytoplasm
CK19	IF, FC	ab52625, Abcam	rabbit	1:200 (IF) 1:400 (FC)	44 KD/Cytoplasm
Goat-anti-rabbit IgG H&L TRITC	IF	ab7051, Abcam	goat	1:400	
Goat-anti-rabbit IgG H&L FITC	IF	ab7050, Abcam	goat	1:400	
Alexa Fluor™ 488 donkey anti-mouse IgG (H+L)	IF	A21202, Invitrogen	donkey	1:400	
Alexa Fluor™ 568 donkey anti-rabbit IgG (H+L)	FC	A10042, Invitrogen	donkey	1:400	
GAPDH-HRP	WB	8884, CST	rabbit	1:1000	37 KD/Cytoplasm
SRSF1	WB	12929-2-AP Proteintech	rabbit	1:1000	32 KD/Nuclei, cytoplasm

SRSF2	WB	ab28428 Abcam	rabbit	1:5000	30 KD/Nuclei, cytoplasm
SRSF3	WB	10916-1-AP Proteintech	rabbit	1:1000	20 KD/Nuclei, cytoplasm
HNRNPA1	WB	11176-1-AP Proteintech	rabbit	1:1000	34 KD/Nuclei
HNRNPA2B1	WB	14813-1-AP Proteintech	rabbit	1:1000	36 KD/Nuclei
HNRNPU	WB	14599-1-AP Proteintech	rabbit	1:1000	120 KD/Nuclei
SYNCRIP	WB	Sc-56703 Santa Cruz	mouse	1:200	70 KD/Nuclei
FUS	WB	Sc-47711 Santa Cruz	mouse	1:200	75 KD/Nuclei
CCNB1	WB	Sc-245 Santa Cruz	mouse	1:500	55 KD/Nuclei, cytoplasm
CDK4	WB	12790S, CST	rabbit	1:1000	30 KD/Nuclei
FKBP51	WB	Sc-271547 Santa Cruz	mouse	1:500	51 KD/Nuclei
c-Myc	WB	ab28428 Abcam	rabbit	1:5000	57 KD/Cytoplasm
c-Myc-HRP	WB	Sc-40 Santa Cruz	mouse	1:500	67 KD/Nuclei, cytoplasm
c-Myc	ChIP/ Co-IP	9402S CST	rabbit	10μL/test	57-70 KD/Nuclei, cytoplasm
Max	Co-IP/ WB	10426-1-AP Proteintech	rabbit	Co-IP: 10μL/test WB: 1:1000	22 KD/cytoplasm
IgG rabbit	CHIP/ Co-IP	Sc-2027x Santa Cruz	rabbit	2μL/test	
IgG mouse	CHIP	Sc-2025 Santa Cruz	mouse	2μL/test	
E2F1	WB	Sc-251 Santa Cruz	mouse	1:500	60 KD/Cytoplasm

Table S6. The primers used in the study.

Primers for PCR and RT-qPCR

Gene Name	Forward primer (5'-3')	Reverse primer (5'-3')
GAPDH	GAGTCAACGGATTTGGTCGT	TTGATTTTGGAGGGATCTCG
SRSF1	CCGCAGGGAACAACGATTG	GCCGTATTTGTAGAACACGTCCT
SRSF2	CCCGATGTGGAGGGTATGAC	GAGACTTCGAGCGGCTGTAG
SRSF3	ATGGAAGAACACTATGTGGCTG	GGGACGGCTTGTGATTTCTCT
MAGOHB	AAGTTTGGGCACGAGTTTCTG	GTCGGCCAACCCTATCAGG
SNRPD1	GAATTGAAGAACGGAACACAGGT	TCCACAAGTAGTGTATCCAGAGG
HNRNPU	GGGGACGGCAAAACAGAACA	AGCACTGAGACGATCTCTTGA
HNRNPA1	TCAGAGTCTCCTAAAGAGCCC	ACCTTGTGTGGCCTTGCAT
HNRNPA2B1	ATTGATGGGAGAGTAGTTGAGCC	AATTCCGCCAACAAACAGCTT
HNRNPD	GCGTGGGTTCTGCTTTATTACC	TTGCTGATATTGTTCCTTCGACA
FUS	ATGGCCTCAAACGATTATACCCA	GTAACTCTGCTGTCCGTAGGG
SYNCRIP	CTGGTCTCAATAGAGGTTATGCG	TCCGGTTGGTGGTATAAAATGAC
CCNB1	AATAAGGCGAAGATCAACATGGC	TTTGTTACCAATGTCCCCAAGAG
FKBP51	CTCCCTAAAATTCCCTCGAATGC	CCCTCTCCTTTCCGTTTGGTT
CDK4	ATGGCTACCTCTCGATATGAGC	CATTGGGGACTCTCACACTCT
MCM-7	GCCTGTGGGAAATATCCCTCG	GTACCACCTGTCGGAACCC

Primers for ChIP assay

Gene Promoter	Forward primer (5'-3')	Reverse primer (5'-3')
E2F1-promoter	TGAGGATGGAAGAGGTGGCT	TTCTGCACGTGACCCTCAAC
HIF1A- promoter	CCGCTAAACACAGACGAGCA	GTTCCTCGAGATCCAATGGC
BCL2- promoter	ACGTGCAACTTGTTTGTCCTCTT	TTGCCTCGTAGCCAATCCTAGT
HNRNPA1-promoter	TCTCCGCCCACTACGCAT	ATTTTTTTGCAATGAACTCTTGTTGAC

HNRNPA2B1-promoter	CCTGTCGCTTGCCACATG	ATGAATTACTCAGCTTAACCATATGTACAAT
HNRNPU-promoter	ACGCGTAGGCCCCATCCAGCTCTAAA	CTCGAGCTGTAACAGTCACTCTTGGAACA
MCM-7-promoter	CCGTCACTCATTCTAGGCCC	AAATTGGCGCGAAACGTC

Primers for dual-luciferase assay

**Gene Promoter**	**Forward primer (5'-3')**	**Reverse primer (5'-3')**
BCL2- promoter	**ACGCGT**CTCCACCTTTGCCTCGTAGC	**CTCGAG**TTTGCACTCGAGCCCTATTAAGT
HNRNPA1-promoter	**ACGCGT**CCGTAGGTTCACTGCCTACTCC	**CTCGAG**ACAGTCAAGTCGCTTGGCAA
HNRNPU-promoter	**ACGCGT**TGACCTGCACTTTAACAGGTCT	**CTCGAG**TATTATTTACACCTTTAAA
MCM-7-promoter	**ACGCGT**GCTCAGAGGTCTTGCTCCTG	**CTCGAG**CTCGGACCGCGGGAAAC

Primers for vector construction

**Cloning primers**	**Forward primer (5'-3')**	**Reverse primer (5'-3')**
c-Myc	CCG**GAATTC**ATGCCCCTCAACGTTAGCT	CGC**GGATCC**TTACGCACAAGAGTTCCG
